# Periphytic Biofilm Formation on Natural and Artificial Substrates: Comparison of Microbial Compositions, Interactions, and Functions

**DOI:** 10.3389/fmicb.2021.684903

**Published:** 2021-07-26

**Authors:** Lingzhan Miao, Chengqian Wang, Tanveer M. Adyel, Jiaqi Zhao, Ning Yan, Jun Wu, Jun Hou

**Affiliations:** ^1^Key Laboratory of Integrated Regulation and Resources Development on Shallow Lakes, Ministry of Education, College of Environment, Hohai University, Nanjing, China; ^2^Centre for Integrative Ecology, School of Life and Environmental Sciences, Deakin University, Melbourne, VIC, Australia

**Keywords:** periphytic biofilms, co-occurrence networks, metabolic functions, artificial and natural substrates, immobilisation

## Abstract

Periphytic biofilms have been widely used in wastewater purification and water ecological restoration, and artificial substrates have been progressively used for periphyton immobilisation to substitute natural substrates. However, there is insufficient knowledge regarding the interaction network structure and microbial functions in biofilm communities on artificial substrates, which are essential attribute affecting their applications in biofilm immobilisation. This study compared the community structure, co-occurrence network, and metabolic functions of bacterial and microeukaryotic periphytic biofilms during a 35-day indoor cultivation on artificial substrates, such as artificial carbon fibre (ACF) and polyvinyl chloride (PVC), and natural substrates, such as pebble and wood. Results demonstrated that different types of artificial substrates could affect the community composition and functional diversity of bacterial and microeukaryotic biofilms. The bacterial and microeukaryotic community on ACF and PVC showed significantly higher Simpson index compared to those on wood. Bacterial networks on artificial substrates were more complex than those on natural substrates, while the keystone species on natural substrates were more abundant, indicating that the bacterial communities on artificial substrates had stronger stability and resistance to external interference. Furthermore, the functional metabolic profiles predicted showed the abilities of bacterial communities to metabolise nitrogen and carbon sources colonised on artificial substrates were stronger than those on natural substrates. These findings demonstrated that artificial substrates could be special niches for microbial colonisation, possibly altering microbial compositions, interactions, and functions. Therefore, this study provides a powerful theoretical basis for choosing suitable artificial substrates for microbial aggregation and immobilisation technology.

## Introduction

Periphyton filtration has been developed to eliminate or decrease pollutants (e.g., organic contaminants, heavy metals, and antibiotics) from natural water environments and wastewater (Matos et al., 2014; Liu et al., 2018a). Periphytic biofilms are complex microorganism communities entwined together by extracellular polymeric substrates (EPS; Douterelo et al., 2018), mainly comprised of bacteria, fungi, microalgae, epiphytes, and detritus (Liu et al., 2018a; Mai et al., 2020). In periphytons, the autotrophic microbial assemblages play a major role in primary productivity and autochthonous carbon production (Calapez et al., 2020). Moreover, heterotrophic communities can effectively biodegrade and remove organic pollutants, promoting the metabolism, mineralisation, and circulation of essential nutrients in aquatic ecosystems (Bondar-Kunze et al., 2016; Mai et al., 2020). Recently, to better utilise the periphytic biofilms’ capacity of biological purification and degradation, various kinds of artificial substrates have been applied to immobilise periphytons, substituting natural substrates (Witt et al., 2011; An et al., 2018). Previous studies demonstrated that periphytic biofilms on artificial substrates were highly heterogeneous and dynamic, and significantly different from those on natural substrates (rock and wood; Miao et al., 2019; Rajeev et al., 2019).

At present, different artificial substrates have been used for periphytic biofilm immobilisation and water purification (Matsumoto et al., 2012; Rajeev et al., 2019). For example, carbon fibres (CFs; An et al., 2018; Liu et al., 2018a), activated CF (ACF; Chen et al., 2013), and polyvinyl chloride (PVC) plastic (Xia et al., 2010; Liu et al., 2014; von Ammon et al., 2018) are widely used as carriers for microorganisms due to the advantages of their physicochemical properties (i.e., strong stability, superior biocompatibility, and large specific surface area). Furthermore, the effects of artificial substrates on periphytons have been analysed in several studies and compared to those on natural substrates, focusing on biomass, microbial diversity, and community structure (Li et al., 2020a; Mai et al., 2020), purification efficiency and the removal mechanisms of periphytons for different contaminants (Matsumoto et al., 2012; Stern et al., 2017; An et al., 2018), and metabolic functions (Boisson and Perrodin, 2006). Most previous studies only evaluated the bacterial communities developed on artificial substrates or natural substrates (Besemer, 2015; Miao et al., 2019; Oberbeckmann and Labrenz, 2020). However, there is still a large knowledge gap regarding microeukaryotic communities such as fungi and metazoan, which have been proved to greatly contribute to the degradation of organic matter in aquatic ecosystems (Amaral-Zettler et al., 2020; Calapez et al., 2020; Starke et al., 2020). The whole biodiversity of the periphyton community directly affects the stable operation and functional characteristics. Hence, it is necessary to better understand the microbial community of periphytic biofilms on different substrates to choose suitable substrates for biofilm immobilisation technology.

Additionally, multispecies in biofilms might exhibit complex microbial communication, interaction, and cooperation, significantly affecting their ecosystem from functioning. The microbial complexity – for example ecological networks – serves as more complex attributes than species richness and community structure, providing deeper information about microbial interaction in biofilms. Recently, seminal studies have used co-occurrence network analysis to explore the symbiotic patterns and pathways among microbial groups in complex communities and their responses to environmental changes (Ma et al., 2016; Liu et al., 2020; Zhang et al., 2020). A set of network topological properties (e.g., average path length, average degree, and modularity) is a way to describe the network co-occurrence patterns and pathways among microbial communities (Cao et al., 2018; Li et al., 2020b). These properties can be used to identify potential microbial interactions and common microbial physiological characteristics (Barberan et al., 2012). Furthermore, topological roles (i.e., connectors, network hubs, module hubs, and peripherals) have been used to reflect the keystone species information in co-occurrence networks, which are of great significance in maintaining the functions of ecosystems (Cao et al., 2018; Li et al., 2020b). However, the interaction network structure in biofilm communities is still overlooked, which is one of the essential attributes affecting their applications in biofilm immobilisation.

Furthermore, microbial community richness, diversity, and co-occurrence networks might not be sufficient to understand how microbial community composition and structure impact ecosystem functions (Yan et al., 2019). This indicates that, in addition to revealing which microbes are on the substrate, it is particularly important to explore the functional characteristics of microbial communities. The difference of metabolic function among organisms is considered to be the basis of environmental change, which is the result of the selection of specific metabolic pathways according to physicochemical conditions (“metabolic niche effects” or “environmental filtration”; Louca et al., 2016). As one of the environmental variables, the substrate type also affects the metabolic pathway of microbes colonised on substrates to a certain extent. Consequently, the metabolic function prediction of bacterial biofilms on different substrates using the functional annotation of Prokaryotic Taxa (FAPROTAX) database can further analyse the biogeochemical cycle process of biofilms in water environment (Louca et al., 2016). Additionally, the fungal trophic mode prediction on different substrates can use FUNGuild database to link the fungal community with function at the guild level (Nguyen et al., 2016).

Here, we hypothesise that artificial substrates may lead to the change of the microbial community structure, the co-occurrence network, and the metabolic function of bacteria and microeukaryotes compared with natural substrates, leading to significant ecological consequences, including changes in the biogeochemical processes and the nutrient cycling of aquatic ecosystems. To test this hypothesis, the source microbial community of Xuanwu Lake was used as the inoculum and two kinds of substrates, i.e., natural (pebble and wood) and artificial (ACF and PVC) were used for indoor periphytic biofilm incubation experiments. The microbial richness, composition, and structure of biofilm communities were compared between natural and artificial substrates. Additionally, the interaction pattern of the co-occurrence network was compared and analysed to explore the ecological impact of artificial substrates on biofilms in aquatic ecosystems. The predicted metabolic profiles for bacteria and fungi were analysed to evaluate the functional characteristics of biofilms on different substrates. In this study, the structure and function of biofilms on different substrates were comprehensively evaluated and compared to provide theoretical support for selecting artificial substrates for biofilm immobilisation.

## Materials and Methods

### Source Microbial Community and Water Collection

The microbial community used as inoculation was detached from pebbles in Xuanwu Lake (32°04'19.7'N and 118°47'9.9'E) using a sterile brush and blade to scrape them down. The biofilms were collected in sterile tanks and stored at −4°C for further experiments. One hundred liters of water were collected in a sterile tank simultaneously and filtered with a 10 μm sieve to remove aquatic organisms and suspended substrates. Triplicates of 500 ml water samples in Xuanwu Lake were transported to the lab, and the water quality parameters was determined (Supplementary Table S1).

### Artificial and Natural Substrates

Artificial carbon fibre and PVC were cut to size 10 cm × 5 cm as the artificial biofilm hanging substrates. At present, ACF is widely used as a material to purify sewage due to its excellent biocompatibility and chemical durability to immobilise microbes (Khatoon et al., 2007; Chen et al., 2013; An et al., 2018). PVC is extensively used in water treatment systems, and several studies have documented the microbial community characteristics of biofilms on PVC (Xia et al., 2010; Janjaroen et al., 2013; Liu et al., 2014; von Ammon et al., 2018). Pebble (diameter 3.0 ± 0.5 cm) and wood (length 10 cm, width 5 cm) were selected as the natural substrates because of the widespread existence of aquatic ecosystems. The formation of functional biofilms on pebble and wood surfaces has also been widely reported (Sailer et al., 2010; Fan and Wang, 2012).

### Periphytic Biofilm Cultivation

Four artificial hydraulic flumes were constructed to simulate the artificial river system (length 4 m, width 0.3 m, and depth 0.3 m), and a detailed description was given in our previous study (Wang et al., 2014). Furthermore, the flow velocity in the flume was kept at 0.1–0.15 m/s, which is approximately the flow velocity in urban rivers (Bondar-Kunze et al., 2016). The substrates mentioned above were used as the biofilm supports for microbial colonisation. To ensure that the specific surface area of biofilm supports had the same order of magnitude, the artificial hydrodynamic flumes were fitted with 60 particles of pebbles and 15 pieces of ACF, PVC, and wood (Supplementary Table S2). The flumes were fed with Xuanwu Lake water pumped by a peristaltic pump. The suspended matter in the raw water was removed by two centrifugal separators and then filtered three times through 90, 20, and 10 μm pore size filters (Wang et al., 2014).

The flumes were placed in the greenhouse and the cooling system kept the water temperature between 17 and 23°C (Graba et al., 2013). The top of the greenhouse was covered with a black cloth that could block about 50% of the solar radiation. Attention was paid to keep the liquid level’s height in the flume constant and adjust the water supply according to the evaporation in time. A Woods Hole culture medium (Supplementary Table S3) was added to the flumes once a week to maintain the normal nutrition level of biofilm growth (Miao et al., 2019). The periphyton biomass (autotrophic and heterotrophic organisms) was evaluated by the determination of dry weight (DW) under different cultivation times (Supplementary Table S4). After 35 days of incubation, the substrates were rinsed with sterile water three times, and the mature periphytic biofilm was collected for further analysis (Nenadović et al., 2015).

### High-Throughput Sequencing

The detailed information of high-throughput sequencing was provided in Supplementary Text S1. Data for biofilm samples are available on the Sequence Read Archive^1^ under the project reference PRJNA736044.

### Data and Statistical Analysis

Alpha diversity was based on the normalized operational taxonomic unit (OTU) abundance table to identify community richness and diversity, comprised of Observed species, Chao1, Shannon, and Simpson indexes, which were calculated with QIIME (V1.9.1; Caporaso et al., 2010) and displayed with Origin2018 (OriginLab Corporation, United States). As for the beta diversity, the difference of microbial community structure between natural and artificial substrates was analysed by principal coordinate analysis (PCoA; Gower and Legendre, 1986; Legendre and Legendre, 1998) and non-metric multidimensional scaling (NMDS) analysis using weighted UniFrac distance matrix (Shepard, 1962) in R (version 3.6.3). Statistical comparison between different biofilm samples was deduced by permutational multivariate ANOVA (PERMANOVA; Anderson, 2005; Zhang et al., 2015).

Comparisons of the abundances of OTUs and functional metabolic pathways of taxonomic microbial communities between the artificial (ACF and PVC) and natural (pebble and wood) substrates were by conducted using Statistical Analysis of Metagenomic Profiles (STAMP; V2.1.3;^2^ Parks and Beiko, 2010; Parks et al., 2014; Yan et al., 2019). Significant differences were determined by Welch’s unequal variances *t*-test, and then multiple tests were performed according to the Benjamini–Hochberg false discovery rate (FDR) procedure (Benjamini and Hochberg, 1995). The *q* values of bacteria were lower than 0.01, and those of microeukaryotes or fungi were lower than 0.05. Comparisons of relative abundances of the most abundant class and metabolic function pathway among the different samples were assessed *via* the heatmap in R (version 3.6.3; Moon, 2016).

A set of network topological properties, i.e., average path length, average degree, modularity, clustering coefficient, and betweenness centrality were calculated using the igraph package in R (version 3.6.3). The co-occurrence network visualization was conducted using Gephi0.9.2.^3^ Principal component analysis (PCA) based on the Bray-Curtis distance (Jolliffe, 1986) was performed using FactoMineR and factoextra packages in R (version 3.6.3). The metabolic predication was analysed using the functional annotation of FAPROTAX database based on the 16S rRNA gene data to explore the biogeochemical cycle functions of microorganisms (Louca et al., 2016).^4^ FUNGuild database was used to analyse the predicted metabolic profiles and evaluate the functional properties of fungi on different substrates (Nguyen et al., 2016).^5^

Six sub-samples were set as parallel samples for biochemical analyses, and the experimental values were represented as the mean ± SD. The alpha diversity indexes, including Observed species, Chao1, Shannon, and Simpson indexes from artificial and natural substrates were compared by one-way ANOVA followed by Tukey’s *post-hoc* tests.

## Results

### Periphyton Biomass

The dynamic growth curve of biofilms on artificial (ACF and PVC) and natural (pebble and wood) substrates is shown in Supplementary Figure S1. The DW of biofilms on pebble was lowest compared to the other three substrates at the late stage of cultivation (Days 28 and 35; ANOVA, value of *p* < 0.05; Supplementary Table S4). After 35 days of incubation, there was a downward trend on the DW of biofilms colonised on PVC, pebble, and wood, which indicated that the biofilm was in the stable mature stage and the attachment and detachment were in a dynamic equilibrium state.

### Alpha and Beta Diversity

The average values of OTUs on ACF, PVC, pebble, and wood in bacterial communities were 4,129, 4,119, 3,998, and 3,877, respectively (Supplementary Figure S2A). Interestingly, the number of unique OTUs in bacterial communities on artificial substrates (ACF and PVC) was lower than that on natural substrates (pebble and wood), which was consistent with the pattern of unique OTUs in microeukaryotic communities (Supplementary Figure S2). Additionally, a total of 737, 703, 749, and 693 OTUs were observed on ACF, PVC, pebble, and wood in microeukaryotic communities, respectively (Supplementary Figure S2B). Although wood samples have the least OTUs, they exhibited the highest number of unique OTUs in microeukaryotic communities.

**Figure 1:**
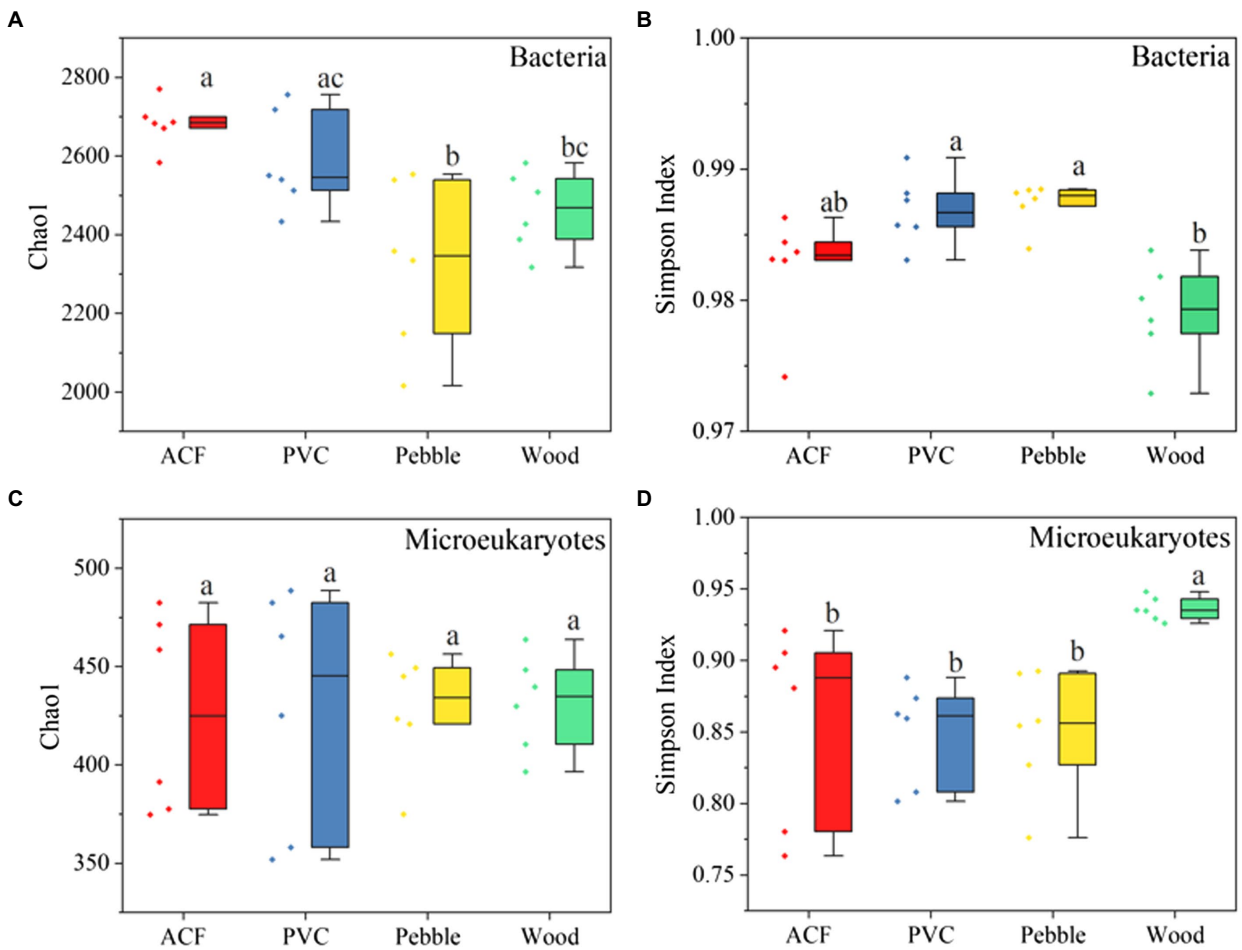
Alpha diversity of bacteria including Chao1 **(A)** and Simpson **(B)** indices and microeukaryotes composed of Chao1 **(C)** and Simpson **(D)** indices, for artificial [artificial carbon fibre (ACF) and polyvinyl chloride (PVC)] and natural (pebble and wood) substrates. The letters represent a significant difference at value of *p* < 0.05 by using one-way ANOVA followed by Tukey’s *post-hoc* tests.

As for the alpha diversity, the bacterial community had significantly higher species richness and diversity compared to the microeukaryotic community (Supplementary Table S5). For bacteria, the biofilm samples from the ACF exhibited higher values of Chao1 index, as shown in Figure 1A, than those on natural substrates (pebble and wood; ANOVA, value of *p* < 0.05). Moreover, the bacterial communities on artificial substrates (ACF and PVC) were more diverse than those on wood (ANOVA, value of *p* < 0.05) as illustrated by the Simpson index (Figure 1B), suggesting that the substrate type had a significant effect on the alpha diversity in bacterial communities.

For microeukaryotes, there was no significant difference among the four substrates in terms of observed species and the Chao1 index (Figure 1C; Supplementary Figure S3). However, the microeukaryotic community on artificial substrates (ACF and PVC) had significantly higher species diversity (Shannon and Simpson indexes) compared to those on wood (ANOVA, value of *p* < 0.05; Figure 1D; Supplementary Figure S3), indicating that the substrate type played a significant role in shaping the diversity of microeukaryotic community structures.

**Figure 2:**
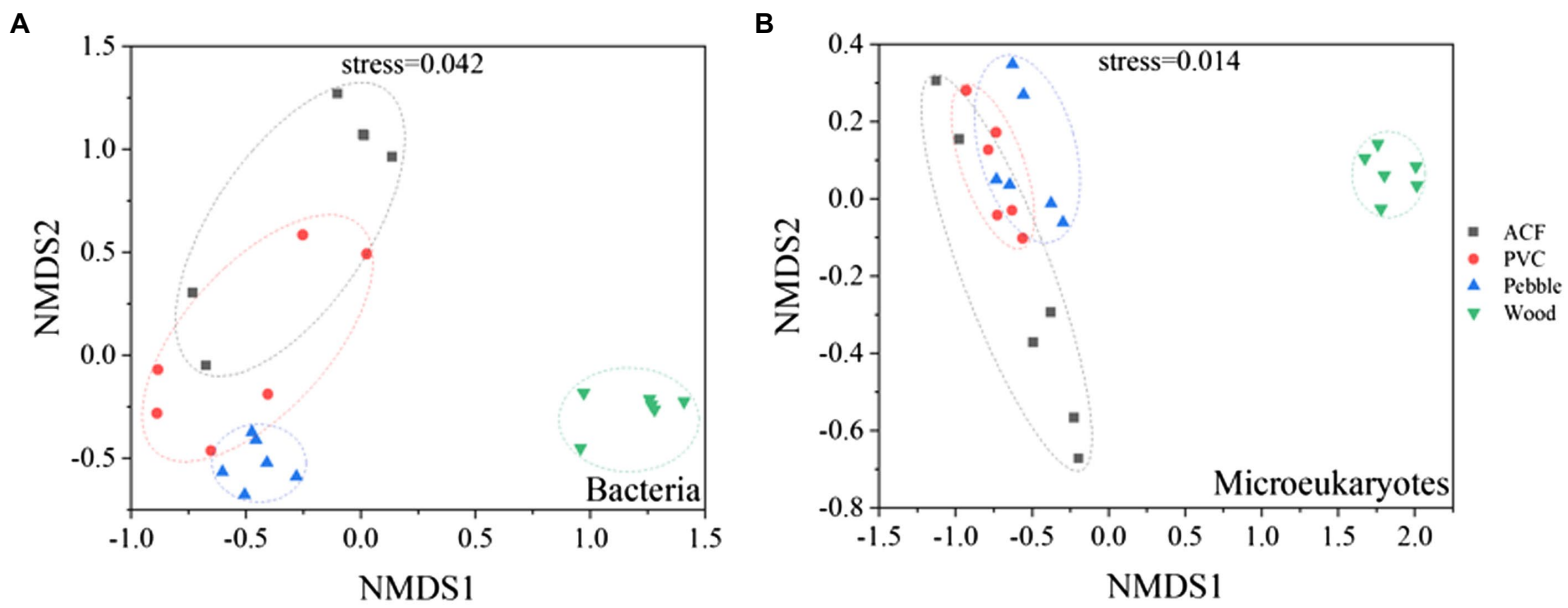
Non-metric multidimensional scaling (NMDS) plots depict bacterial **(A)** and microeukaryotic **(B)** biofilms on artificial (ACF and PVC) and natural (pebble and wood) substrates using the phylogenetically weighted UniFrac distance matrix. Statistical comparison between different samples was deduced by permutational multivariate ANOVA (PERMANOVA), and results are provided in Supplementary Table S6.

The analysis of beta diversity was shown in Figure 2. For the bacterial communities, there were significant differences among the four substrates by pairwise comparison (values of *p* < 0.01, both Adonis and Anosim; Supplementary Table S6). Furthermore, the bacterial biofilms on artificial substrates (ACF and PVC) and wood were apparently separated primarily along the first coordinate axis (Figure 2A). Nevertheless, the bacterial communities on artificial substrates (ACF and PVC) and pebble were apparently separated mainly along the second coordinate axis (Figure 2A), which were consistent with PCoA (Supplementary Figure S4). Additionally, according to the Adonis (*F* = 4.9, *R*^2^ = 0.18, *p* = 0.006) and Anosim analyses (*p* = 0.003, *R* = 0.30), there were significant differences between artificial and natural substrates (Supplementary Table S6).

For the microeukaryotic communities, the microeukaryotic biofilms on the four substrates were conspicuously separated primarily along the first coordinate axis, according to the order of ACF, PVC, pebble, and wood (Figure 2B). Moreover, significant differences had been found between artificial and natural substrates, as illustrated by the Adonis (*F* = 8.42, *R*^2^ = 0.28, *p* = 0.002) and Anosim analyses (*p* = 0.001, *r* = 0.41; Supplementary Table S6).

### Biofilm Community Composition and Structure

The detailed information of biofilm community composition and structure was provided in Supplementary Text S2, Supplementary Figures S5–8, and Supplementary Tables S7–9.

### Co-Occurrence Network Analysis

**Figure 3:**
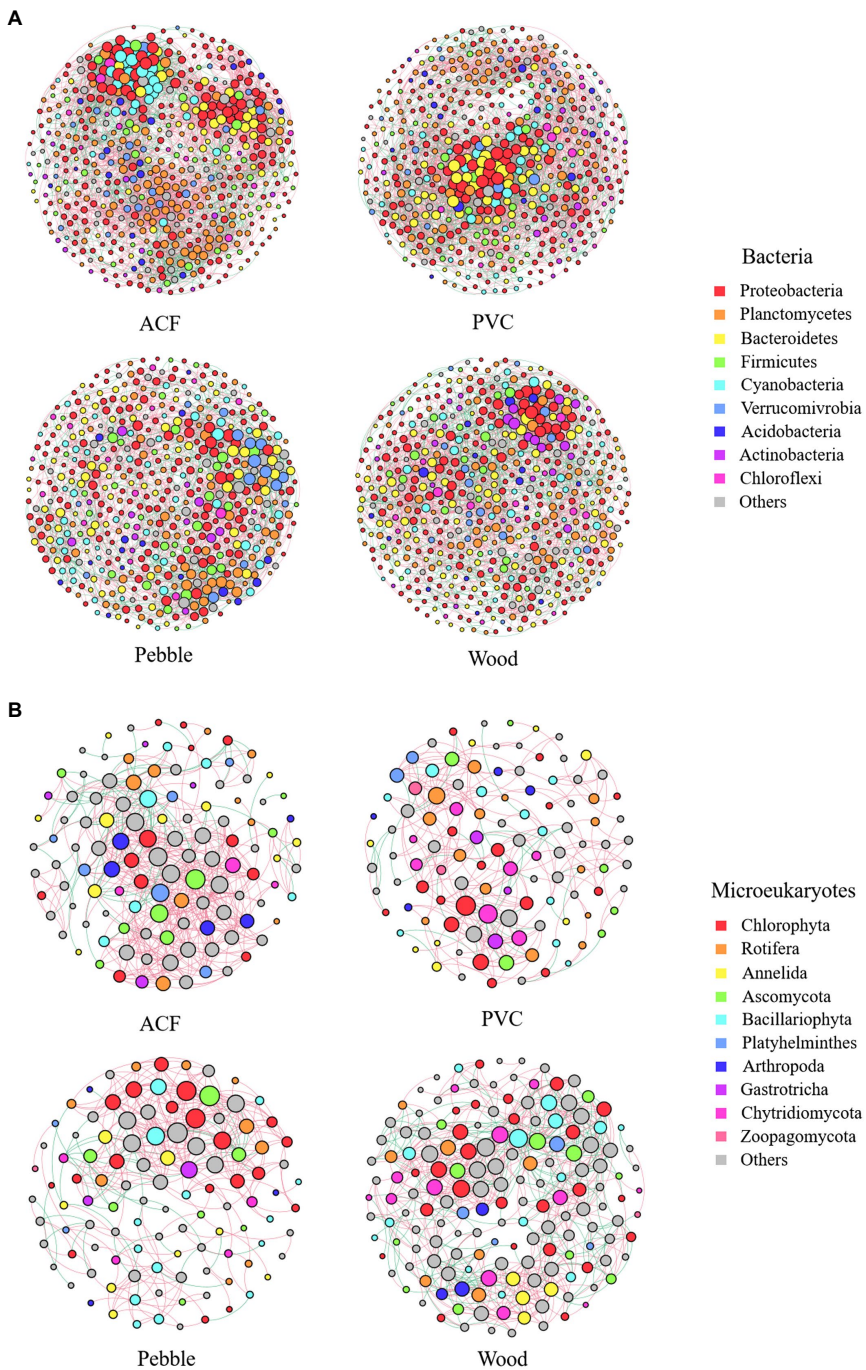
Networks of bacteria **(A)** and microeukaryotes **(B)** at phylum level for the biofilm samples colonised on artificial (ACF and PVC) and natural (pebble and wood) substrates. Each point represents an independent bacterial or microeukaryotic operational taxonomic unit (OTU), node size is proportional to the relative abundance, red edges represent positive relationships, and green edges represent negative relationships among the OTUs in the network.

To better understand the manifestation of biological interrelationships in microbial community aggregation and identify potential keystone species, a microbial co-occurrence network analysis (phylum level) of periphytic biofilms colonised on natural (pebble and wood) and artificial (ACF and PVC) substrates was constructed based on significant correlations. Due to significant differences in community composition and structure, eight networks were constructed for bacteria and microeukaryotes colonised on natural (pebble and wood) and artificial (ACF and PVC) substrates (Figure 3). The proportions of nodes of the most common phylum of bacteria and microeukaryotes in the network for the four substrates were shown in Supplementary Table S10. Additionally, key topological features of bacterial and microeukaryotic networks of periphytic biofilms colonised on natural (pebble and wood) and artificial (ACF and PVC) substrates were listed in Supplementary Table S11.

In general, bacterial networks (Figure 3A) were more complex and cooperative than microeukaryotic networks (Figure 3B), exhibiting more nodes and edges. For bacterial networks, the nodes of biofilms on natural (pebble and wood) and artificial (ACF and PVC) substrates mainly belonged to the phyla Proteobacteria, Bacteroidetes, Planctomycetes, Cyanobacteria, and Firmicutes. The bacterial networks of biofilms on artificial substrates had higher proportions of Planctomycetes than those on natural substrates. Nevertheless, the bacterial networks of biofilms on artificial substrates had lower proportions of Bacteroidetes than those on natural substrates (Supplementary Table S10). For microeukaryotic networks, the nodes belonging to the phyla Chlorophyta, Bacillariophyta, and Rotifera accounted for a large proportion. The microeukaryotic networks of biofilms on artificial substrates had higher proportions of Annelida, Arthropoda, Gastrotricha, and Rotifera than those on natural substrates. However, the microeukaryotic networks of biofilms on artificial substrates had lower proportions of Chlorophyta than those on natural substrates (Supplementary Table S10).

Furthermore, the co-occurrence network of bacteria on ACF captured 5,306 edges among 547 nodes, which was the most closely connected community compared with the network on the other three substrates (Supplementary Table S11). However, the co-occurrence network of microeukaryotes on wood showed a closer connection. These findings illustrated that the substrate type could affect the biological interaction network on bacteria and microeukaryotes. Collectively, for bacteria, the networks on artificial substrates had higher positive edges, average clustering coefficient, average degree and graph density, and lower average path length than those on natural substrates, which indicated that the bacterial networks on artificial substrates were more complex and connected than those on natural substrates. For microeukaryotes, the network on wood had the lowest positive edges and highest average degree compared to the other three substrates. Furthermore, the network on ACF had the lowest average path length and network diameter and highest average clustering coefficient and graph density compared to the other three substrates. Due to the high modularity values (greater than 0.5), networks of bacteria and microeukaryotes on different substrates except pebble had modular structures (Supplementary Table S11).

**Figure 4:**
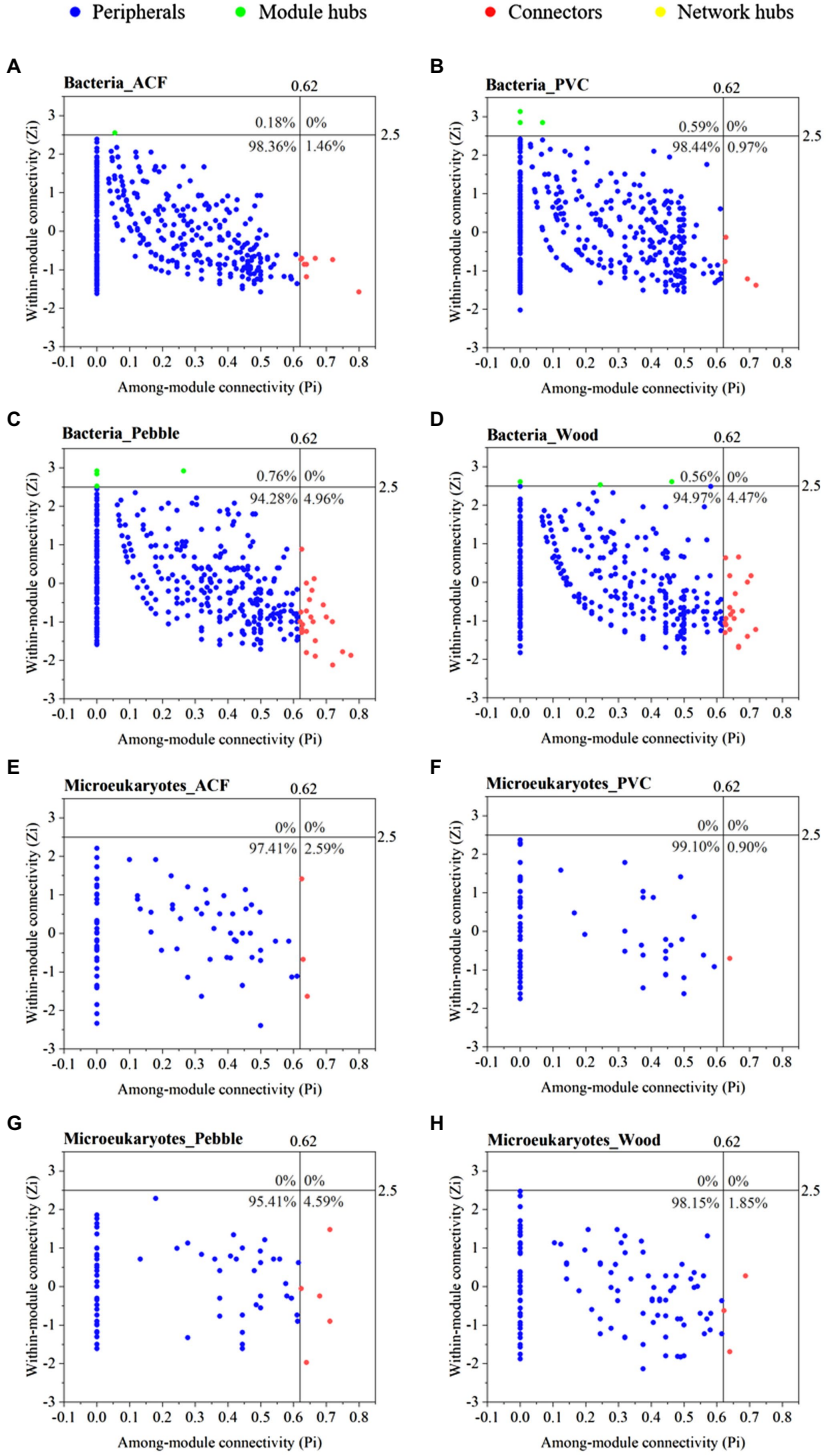
Zi-Pi plot of bacteria (**A**–**D**) and microeukaryotes (**E**–**H**) at OTU level for the biofilm samples colonised on artificial (ACF and PVC) and natural (pebble and wood) substrates. The topological role of each OTU was determined according to the scatter plot of within-module connectivity (Zi) and among-module connectivity (Pi). Connectors, network hubs, and module hubs were selected as keystone species, and the annotation information of keystone species was shown in Supplementary Table S12.

The topological roles of the OTUs identified in these eight networks were shown as a Zi-Pi plot (Figure 4). According to the scatter diagram of within-module connectivity (Zi) and among-module connectivity (Pi), topologically, the topological roles of each OTU in the network were determined as connectors, module hubs, network hubs, and peripherals, respectively (Cao et al., 2018). Ecologically, connectors and module hubs represented generalists, while network hubs indicated supergeneralists, and peripherals represented specialists (Li et al., 2020b). In this study, connectors, module hubs, and network hubs were selected as keystone species (Supplementary Table S12). Their extinction might lead to community fragmentation, where the impact was due to biological interactions rather than high abundance. Generally, there was no repetition of the same keystone species in these eight networks, which indicated that each network had its own functional heterogeneity and specificity (Supplementary Table S12).

In the bacterial network, the proportions of connectors were more on natural substrates (pebble and wood) than artificial (ACF and PVC; Figures 4A–D). More interestingly, the proportions of connectors on pebble were most abundant both on bacterial and microeukaryotic networks, and the connectors in bacterial networks belonged to phyla Proteobacteria (8/25), Cyanobacteria (6/25), and Planctomycetes (5/25; Supplementary Table S12). Furthermore, there was no network hub in the bacterial networks, indicating that there was no OTU with a high connection inside and outside the modules in these biological networks (Figures 4A–D). More importantly, the proportions of module hubs on PVC, pebble, and wood were higher than on ACF in bacterial networks, demonstrating that the interaction between bacteria on the former was mostly produced in the module, and the interaction ability was stronger than on ACF (Figures 4A–D).

In addition, no module and network hub roles have been found in the microeukaryotic networks, indicating that the connectivity within and outside each module in microeukaryotic networks was low (Figures 4E–H). In microeukaryotic networks, connectors were more common on pebble, which indicated that the community connectivity of microeukaryotic microorganisms on pebble was stronger than other substrates (Figures 4E–H). Furthermore, OTU 18, OTU 19, and OTU 191 derived from the phylum Chlorophyta were connectors on the pebble-associated network (Supplementary Table S12).

### Biofilm Metabolic Functional Prediction

**Figure 5:**
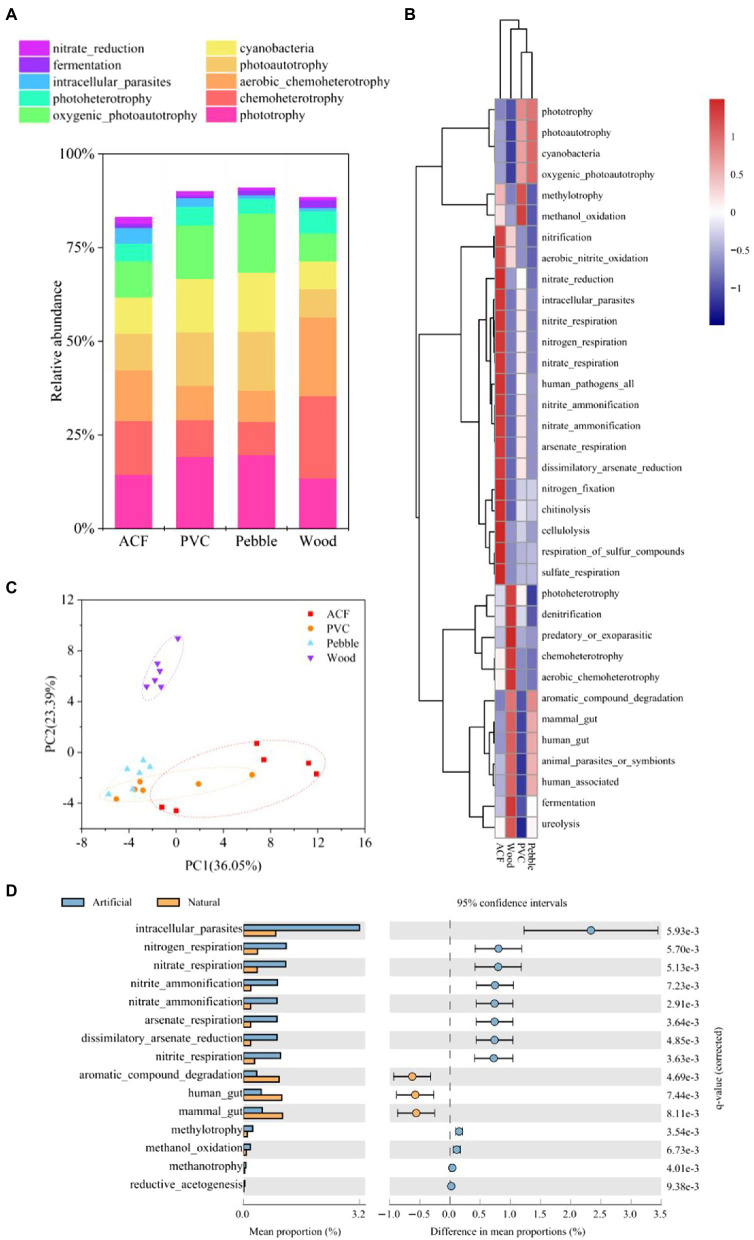
**(A)** Relative abundance of the top 10 most abundant metabolic function pathways in bacterial biofilms from artificial (ACF and PVC) and natural (pebble and wood) substrates. Statistical comparison between the substrate types was performed using one-way ANOVA followed by Tukey’s *post-hoc* tests, and results FIGURE 5 | are provided in Supplementary Table S13. **(B)** Heatmap-mean analysis of bacterial biofilms at the function level (*n* = 6). **(C)** Principal component analysis (PCA) plot depicts bacterial biofilms on artificial (ACF and PVC) and natural (pebble and wood) substrates based on the Bray-Curtis distance. Statistical comparison was deduced by PERMANOVA, and results are provided in Supplementary Table S14. **(D)** Comparison of the bacterial metabolic function pathways’ abundance between artificial (ACF and PVC) and natural (pebble and wood) substrates using Statistical Analysis of Metagenomic Profiles (STAMP). See also Supplementary Table S15.

The FAPROTAX database was used to map prokaryotic clades to metabolic functions based on the 16S rRNA gene data (Louca et al., 2016; Li et al., 2019). The top 10 predominant bacterial metabolic activities in biofilms on different substrates were identified (Figure 5A). The metabolic activities of biofilms on ACF were dominated by phototrophy (14.40%), chemoheterotrophy (14.37%), and aerobic chemoheterotrophy (13.52%); phototrophy (19.21%), photoautotrophy (14.33%), and cyanobacteria (14.26%) in bacterial biofilms on PVC; phototrophy (19.68%), photoautotrophy (15.77%), and cyanobacteria (15.73%) in bacterial biofilms on pebble; and chemoheterotrophy (21.99%), aerobic chemoheterotrophy (20.97%), and phototrophy (13.40%) in bacterial biofilms on wood, respectively (Figure 5A). These results indicated that chemoheterotrophy, aerobic chemoheterotrophy, and phototrophy were the most dominant metabolic functions in bacterial biofilms on ACF and wood, while phototrophy and photoautotrophy were the most dominant metabolic functions in bacterial biofilms on PVC and pebble.

Interestingly, bacteria on ACF had powerful nitrogen cycle functions (i.e., nitrification, nitrite/nitrate respiration, nitrite/nitrate ammonification, and nitrogen fixation). Moreover, bacterial biofilms on ACF were involved in the arsenic and sulfur cycle and had unique functions (i.e., chitinolysis and cellulolysis; Figure 5B). In the metabolic pathways of bacterial biofilms on wood, there was a high correlation with heterotrophic metabolism, including photoheterotrophy and chemoheterotrophy (Figure 5B). Furthermore, due to the high correlation with phototrophy and methylotrophy, bacterial biofilms on PVC played an important role in the carbon cycle, including the synthesis and decomposition of carbonaceous organic compounds (Figure 5B). Moreover, the metabolic functions of bacterial biofilms on pebble were not only related to photoautotrophy and cyanobacteria, but also to mammals and humans (Figure 5B). Unsurprisingly, bacterial biofilms on these four substrates had common and unique metabolic functions, which might be caused by complex environment variables, such as substrate characteristics, water quality conditions, and planktonic microbial communities in water, etc. In addition, according to PCA, the distribution of biofilm metabolic functions colonised on different substrates was partially overlapped, which might be due to some similar functions (Figure 5C; Supplementary Table S14).

Furthermore, the effect of substrate types (artificial and natural substrates) on bacterial metabolic functions was analyzed by STAMP. As illustrated in Figure 5D, a total of 15 representative metabolic functions were screened and there were significant differences between natural and artificial substrates (value of *q* < 0.01; Supplementary Table S15). Among the 15 metabolic function pathways, only three functions [i.e., human gut, mammal gut, and aromatic compound degradation on natural substrates (pebble and wood)] were stronger than those on artificial substrates (ACF and PVC). Furthermore, the abilities of bacterial biofilms to metabolise nitrogen, carbon, and arsenic sources colonised on artificial substrates were stronger than those on natural substrates (Figure 5D; Supplementary Table S15).

For microeukaryotes, no similar metabolic function prediction tools were available to classify the microeukaryotic community into metabolic functional groups. Therefore, we chose fungi belonging to the microeukaryotic community as the research object and used FUNGuild database to link the fungal community with function at the guild level for fungal trophic mode prediction (Nguyen et al., 2016; Wei et al., 2018). According to the classification of FUNGuild, fungi could be divided into three types: pathotroph, saprotroph, and symbiotroph. The trophic modes of fungi on PVC were lichenized and ectomycorrhizal, both belonging to symbiotroph (Supplementary Figure S9). Additionally, arbuscular mycorrhizal and soil saprotroph were distinctive nutrition modes of fungi on ACF. However, saprotroph, such as wood saprotroph and plant saprotroph, was the main fungal trophic mode of biofilms on pebble. Moreover, a plant pathogen belonging to pathotroph was a unique nutrition mode of fungi on wood. Unsurprisingly, there was no significant fungal trophic mode difference between natural and artificial substrates (value of *q* < 0.05; Supplementary Figure S9; Supplementary Table S15) except the plant pathogen (value of *q* = 0.015).

## Discussion

This study comprehensively compared the community structure and co-occurrence network of bacterial and microeukaryotic periphytic biofilms on artificial (ACF and PVC) and natural (pebble and wood) substrates. We demonstrated that different artificial substrates could affect the community structure and composition diversity of bacterial and microeukaryotic biofilms. Furthermore, bacterial networks on artificial substrates were more complex than on natural substrates, but the keystone species on natural substrates were more abundant. More importantly, the substrate type could impact metabolic functions of biofilms, thus affecting the biogeochemical cycle of aquatic ecosystems. These results suggest that artificial substrates can be special niches for microbial colonisation, possibly altering microbial community compositions and community interaction and thereby changing microbial metabolic functions. Therefore, our study provides a good theoretical basis for choosing suitable substrates for microbial aggregation and immobilisation technology.

### Effects of Substrate Types (Artificial and Natural) on the Microbial Compositions and Interactions

The substrate properties, such as surface texture, surface roughness, hydrophobicity, and biocompatibility, were identified as selective factors of microbial colonisation and biofilm formation on different substrate types (Chen et al., 2013; von Ammon et al., 2018; Miao et al., 2020a). In our previous study, we found that PVC has strong hydrophobicity and large surface roughness (Miao et al., 2020a). Unsurprisingly, surface roughness and texture of the substrate could provide more attachment points for biofilms (Parrish and Fahrenfeld, 2019; Miao et al., 2020b). Moreover, bacteria adhere better to hydrophobic surface than to hydrophilic surface (Ogonowski et al., 2018). A recent study found that the bacterial communities on PVC were mainly dominated by Proteobacteria (85–97%) and Bacteroides (2–10%) cultured in coral areas (Feng et al., 2020). Liu et al. (2014) found that Proteobacteria (greater than 80%) was the most dominant group in the PVC biofilm. Moreover, our study found that the bacterial communities on PVC were primarily composed by Proteobacteria (average 31.25%) and Planctomycetes (average 18.97%; Supplementary Table S6). These findings demonstrated that Proteobacteria was easily colonised on PVC surface and became the most advantageous phylum. Several studies have documented that carbon fibre was widely applied for biofilm supports owing to its high chemical durability, biocompatibility, and good capacity to immobilise microbes (An et al., 2018; Liu et al., 2018b). Chen et al. (2013) have studied the functional group characteristics of EPS in biofilms on ACF substrate. In the present study, the average clustering coefficient and average degree were highest in the bacterial and microeukaryotic networks on ACF, indicating that there were more groups of species with similar functions on ACF. This finding might be related to its good biocompatibility and large surface area.

Co-occurrence network analysis has been used to study the possible interactions between microorganisms (Ma et al., 2016; de Vries et al., 2018; Liu et al., 2020). The topological characteristics of the network could reflect the connectivity and interaction between microorganisms (Chen et al., 2020). In the present study, the networks on artificial substrates had a higher average clustering coefficient, average degree and graph density, and lower average path length than those on natural substrates in the bacterial networks, indicating that there were more groups of species with similar functions on artificial substrates that tended to live in collective cooperation. It is generally believed that the dominant population in the environmental microbial community plays an important role in the community (Zhao et al., 2020). In networks, positive and negative interactions (edges) were considered mutualistic and antagonistic relationships between microbial species (Xu et al., 2017). In addition, cross-feeding of metabolic by-products, co-aggregation, co-colonisation, and niche overlap could cause positive correlations (Widder et al., 2016; Li et al., 2020b). In our present study, the bacterial networks on artificial substrates had higher positive edges than those on natural substrates, which might be due to the more intense mutualistic and antagonistic relationships of bacteria on artificial substrates. Furthermore, networks of bacteria and microeukaryotes on different substrates except pebble had high modularity values (greater than 0.5; Supplementary Table S11), which indicated the niche differentiation or functional unit diversity of microbial communities (Li et al., 2020b).

Ecologically, peripherals represent specialists while module hubs and connects represent generalists and network hubs represent supergeneralists. Module hubs, connectors, and network hubs could be seen as keystone species (Cao et al., 2018; Li et al., 2020b). Their extinction might lead to community fragmentation, an effect due to biological interactions rather than high abundance (Li et al., 2020b). In our study, the proportions of connectors were more on natural substrates (pebble and wood) than artificial substrates (ACF and PVC; Figure 4). More interestingly, the proportions of connectors on pebble were most abundant both on bacterial and microeukaryotic networks, indicating that the substrate type might affect the connectivity of biological network structures. The increase of the complexity of network structure, such as the abundance of key species, would enhance the stability of mixed interaction communities, which was an important performance for maintaining the biodiversity of ecological communities (Li et al., 2020b). Consequently, it is necessary to better understand the co-occurrence ecological network of microorganisms colonised on artificial substrates because of the differentiation of network patterns on natural substrates.

### Effects of Substrate Types (Artificial and Natural) on the Microbial Functions

Heterotrophic bacteria are often used as decomposers, responsible for *in situ* remediation and degradation of organic matter in aquatic ecosystems (Yan et al., 2019). In the present study, chemoheterotrophy and aerobic chemoheterotrophy were the most dominant biogeochemical cycle functions in bacterial biofilms on ACF and wood (Figure 5B). Furthermore, most denitrifying bacteria are heterotrophic bacteria, which use organic matter as a nitrogen source and energy source for anaerobic respiration (Patel et al., 2014). More importantly, denitrification is the principal pathway of nitrogen metabolism, and the predominant denitrifying bacteria are mainly associated with *Alphaproteobacteria*, *Betaproteobacteria*, *Gammaproteobacteria*, and *Flavobacteriia* (Tian and Wang, 2021). In our study, the relative abundances of *Gammaproteobacteria* (23.97%) and *Flavobacteriia* (2.68%) were significantly overrepresented on wood compared with the other three substrates (Supplementary Table S8). Additionally, because of the oxygenic-anaerobic environment in periphytic biofilms, nitrifying and denitrifying microorganisms could colonise and grow on them (Yan et al., 2019). Surprisingly, bacterial biofilms on ACF had powerful nitrogen cycle functions (Figure 5B). These findings demonstrate that the substrate type might affect the microbial attachment and colonisation, as well as change the microenvironment on biofilms, thus affecting the metabolic function of biofilms.

Carbon metabolism is an important part of the microbial metabolic network and includes the extremely significant mechanism of energy biosynthesis, providing carbon sources and energy for nitrogen metabolism (Tian and Wang, 2021). In the present study, phototrophy and photoautotrophy were the most dominant metabolic functions in bacterial biofilms on PVC and pebble. A similar study demonstrated that the degradation ability of Bacteroidetes to cellulose, chitin, and pectin made their ecological significance in marine ecosystems increasingly prominent. Similarly, bacterial biofilms on ACF had unique functions (i.e., chitinolysis and cellulolysis; Figure 5B) because of their high relative abundance of Bacteroidetes (16.56%; Supplementary Table S7). Furthermore, the abilities of bacterial biofilms to metabolise nitrogen, carbon, and arsenic sources colonised on artificial substrates were stronger than on natural substrates (Figure 5D; Supplementary Table S15). Although fungi have high phylogenetic diversity and diverse metabolic potential, they have received little attention in biofilm colonisation on different substrates. Fungi can play a variety of ecological roles (e.g., decomposers of organic matter, predators, endophytes, or pathogens; Wang et al., 2020). In our study, the trophic mode of fungi on PVC was symbiotroph, and saprotroph was the main fungal trophic mode of biofilms on pebble. Consequently, the type of substrate could affect the metabolic performance and the metabolism of nutrients of the colonised microbial communities.

## Conclusion

This study highlights the remarkable potential of immobilised periphytic biofilms on artificial substrates (ACF and PVC). The present study demonstrated that:

- Distinct communities of bacteria and microeukaryotes colonised on artificial substrates exhibit various microbial community structures and compositions compared with those colonised on natural substrates (pebble and wood).
- A differentiation of network patterns and keystone species are found between natural and artificial substrates.
- Abilities of bacterial biofilms to metabolise nitrogen and carbon sources colonised on artificial substrates are stronger than those on natural substrates.

Therefore, this study emphasized the effect of substrate types (artificial and natural substrates) on biofilm community compositions, interactions, and functions, which could further affect their application of biofilm immobilisation. With the application of artificial substrates to immobilise periphytons, the potential impacts of microbial aggregations, interactions, and functions should be further studied, thus expanding our understanding of the mechanism of biofilm formation, the specificity of biofilm community structures and network relationships, and community metabolic functions on artificial substrates.

## Data Availability Statement

The raw data supporting the conclusions of this article will be made available by the authors, without undue reservation.

## Author Contributions

LM and CW: conceptualization, methodology, software, investigation, and writing – original draft. TA and JZ: writing – review and editing, and data curation. NY and JW: validation, formal analysis, visualization, and software. JH: resources, writing – review and editing, and supervision. All authors contributed to the article and approved the submitted version.

## Conflict of Interest

The authors declare that the research was conducted in the absence of any commercial or financial relationships that could be construed as a potential conflict of interest.

## Publisher’s Note

All claims expressed in this article are solely those of the authors and do not necessarily represent those of their affiliated organizations, or those of the publisher, the editors and the reviewers. Any product that may be evaluated in this article, or claim that may be made by its manufacturer, is not guaranteed or endorsed by the publisher.

## Footnotes

^1^http://www.ncbi.nlm.nih.gov/sra/

^2^http://kiwi.cs.dal.ca/Software/STAMP

^3^http://gephi.github.io/

^4^http://www.loucalab.com/

^5^http://www.funguild.org/
