# Supplementary material for: Periphytic Biofilm Formation on Natural and Artificial Substrates: Comparison of Microbial Compositions, Interactions, and Functions
Source: Front Microbiol. 2021 Jul 26;12:684903. doi: 10.3389/fmicb.2021.684903 (PMC8350161; doi:10.3389/fmicb.2021.684903)
Supplement: Supplementary file 1 [file Data_Sheet_1.docx]

**Supplementary materialof**

**Periphytic biofilm formation on natural and artificial substrates: Comparison of microbial compositions, interactions and functions**

Lingzhan Miao^1^, Chengqian Wang^1^, Tanveer M. Adyel^2^, Jiaqi Zhao^1^, Ning Yan^1^, Jun Wu^1^, Jun Hou*^1^

^1^Key Laboratory of Integrated Regulation and Resources Development on Shallow Lakes, Ministry of Education, College of Environment, Hohai University, Nanjing, People’s Republic of China, 210098

^2^Centre for Integrative Ecology, School of Life and Environmental Sciences, Deakin University, Melbourne, VIC 3125, Australia

*Corresponding author:

Professor Jun Hou, Tel.: +86-25-83787930; fax: +86-25-83787930.

Author affiliation: Xikang Road 1^st^, Nanjing, People’s Republic of China, 210098

E-mail: [hhuhjyhj@126.com;](mailto:hhuhjyhj@126.com;)[hjy_hj@hhu.edu.cn](mailto:hjy_hj@hhu.edu.cn)

**Supplementary Text S1: High-throughput sequencing**

The mature biofilms colonized on artificial and natural substrates were sequenced with high throughput (Illumina HiSeq 2500 platform, Guangdong Magigene Biotechnology Co. Ltd., China) to explore the composition and function of bacterial and microeukaryoticcommunities. The high-throughput sequencing method mainly refers to the high-throughput sequencing method manual provided by the Guangdong Magigene Biotechnology Co. Ltd., China.

Text S1.1 Extraction of genome DNA

Approximately 0.5 g of each biofilm sample were collected for bioinformatics analysis. DNA was extracted using DNA extraction kit(Omega Bio-tek, Norcross, GA, US)for the corresponding sample. DNA integrity and purity were monitored on 1% agarose gels. Meanwhile, DNA concentration and purity were measured using the NanoDrop One (Thermo Fisher Scientific, MA, USA). Using the genome DNA as a template, PCR was amplified using primers with barcode and Premix Taq (TaKaRa) from the selection of sequencing areas.

Text S1.2Amplicon Generation

16S rRNA genes of V4 region were amplified using primer (806R/515F) to identify the diversity of bacteria. 18S rRNA genes of V4 region were amplified using primer (706R/528F) to identify the diversity of microeukaryotes.Primers were synthesized by Invitrogen (Invitrogen, Carlsbad, CA, USA).PCR reactions, containing 25μl 2x Premix Taq (Takara Biotechnology, Dalian Co. Ltd., China), 1μl each primer(10mM) and 3μl DNA (20ng/μl) template in a volume of 50 µl, were amplified by thermocycling: 5min at 94°C for initialization; 30 cycles of 30s denaturation at 94°C, 30s annealing at 52°C, and 30s extension at 72°C; followed by 10 min final elongation at 72°C. Three replicates per-sample and each PCR products of the same sample were mixed, the PCR instrument was BioRad S1000 (Bio-Rad Laboratory, CA, USA)

Text S1.3 PCR Products Detection, Pooling and Purification

The length and concentration of the PCR product were detected by 1% agarose gel electrophoresis. Samples with bright main stripcan be uesd for further experiments. PCR products was mixed in equidensity ratios according to the GeneTools Analysis Software (Version4.03.05.0, SynGene). Then, mixture PCR products was purified with EZNA Gel Extraction Kit (Omega, USA).

Text S1.4 Library preparation and sequencing

Sequencing libraries were generated using NEBNext® Ultra™ DNA Library Prep Kit for Illumina® (New England Biolabs, MA, USA) following manufacturer's recommendations and index codes were added. The library quality was assessed on the Qubit@ 2.0 Fluorometer (Thermo Fisher Scientific, MA, USA) and Agilent Bioanalyzer 2100 system (Agilent Technologies, Waldbron, Germany). At last, the library was sequenced on an IlluminaHiseq2500 platform and 250 bp paired-end reads were generated.

Text S1.5 Sequencing data processing

Quality filtering on the paired-end raw reads were performed under specific filtering conditions to obtain the high-quality clean reads according to the Trimmomatic(V0.33, http://www.usadellab.org/cms/?page=trimmomatic) quality controlled process. Then, paired-end clean reads were merged using FLASH(V1.2.11, https://ccb.jhu.edu/software/FLASH/) according to the relationship of the overlap between the paired-end reads, when at least 10 of the reads overlap the read generated from the opposite end of the same DNA fragment, the maximum allowable error ratio of the overlap region of 0.1, and the spliced sequences were called Raw Tags. Sequences were assigned to each sample based on their unique barcode and primer using Mothur software (V1.35.1, http://www.mothur.org), after which the barcodes and primers were removed and got the effective Clean Tags.

Text S1.6 OTU cluster and Species annotation

Sequence’s analysis were performed by usearch software (V8.0.1517, http://www.drive5.com/usearch/). Sequences with ≥97% similarity were assigned to the same OTU. An OTU is thought to possibly represent a species. The most frequently occurring sequence was extracted as representative sequence for each OTU and was screened for further annotation. During the clustering, usearch can remove both the chimera sequence and singleton OTU. For each representative sequence, the silva (for 16S, 18S, chloroplast and mitochondria, self-organized, https://www.arb-silva.de/)database was used to annotate taxonomic information (set the confidence threshold to default to ≥0.5). Removes the OTU and its Tags, which are annotated as chloroplasts or mitochondria (16S amplicons) and can’t be annotated to the kingdom level. Then get the number of effective tags (No. of seqs) and OTU taxonomy synthesis information table (otu_table) for the final analysis. In order to study phylogenetic relationship of different OTUs, the KRONA software (http://sourceforge.net/projects/krona/) was used to visualizes the results of individual sample annotations. In order to quickly and intuitively study the species composition and abundance informationin the sample, the GraPhlAn software (http://huttenhower.sph.harvard.edu/graphlan) was used to get a single sample OTU annotation circle graph.In order to study the difference of the dominant species in different groups, the OTU representative sequence with the relative abundance in the first 50 and annotated to the level of the genus was selected, multiple sequence alignment was conducted using the FastTree software, and the relative abundance of each OTU and the species annotation information of the representative sequence were combined with the ggtree software package for visual display. After that, OTU abundance information were normalized using a standard of sequence number corresponding to samples**.** Subsequent analysis of alpha diversity and beta diversity were all performed basing on this output normalized data.

**Supplementary Text S2: Biofilm community composition and structure**

In general, the substrate type has a significant influence on the composition and structure of microeukaryotic and bacterial communities. At the phylum level, the bacterial communities of all biofilm samples were dominated by Proteobacteria (28.93–49.28%), Planctomycetes (9.58–23.40%), Cyanobacteria (8.01–22.67%) and Bacteroidetes (14.29–16.56%) (SupplementaryFigure S5A and SupplementaryTable S7). The phyla Proteobacteria, Planctomycetes, Chlorobi and Chloroflexi were significantly different between artificial (ACF and PVC) and natural (pebble and wood) substrates (ANOVA, P-value < 0.01) (SupplementaryTable S7). Furthermore, the relative abundances of Planctomycetes and Chlorobi were significantly overrepresented on artificial substrates compared with natural substrates (ANOVA, P-value < 0.01) (SupplementaryTable S7).

For microeukaryotes, the relative abundances of kingdoms were followed by Eukaryota, Metazoa, Fungi and Viridiplantae. At the phylum level, the microeukaryotic communities of all biofilm samples on different substrates were dominated by Annelida (3.37–45.97%), Chlorophyta (4.94–17.62%), Platyhelminthes (2.48–12.24%) and Chytridiomycota (4.84–9.86%) (SupplementaryFigure S5B and SupplementaryTable S7). In addition, Bacillariophyta and Eustigmatophyceae belonging to Eukaryota were more enriched on the natural substrates (ANOVA, P-value < 0.001), while Annelida, Arthropoda and Gastrotricha belonging to Metazoa were higher on artificial substrates (ANOVA, P-value < 0.005).

At the class level, there was no significant difference in the relative abundances of Alphaproteobacteria and Betaproteobacteria in bacterial communities between artificial (ACF and PVC) and natural (pebble and wood) substrates (SupplementaryFigure S6A and SupplementaryTableS8). Nevertheless, Gammaproteobacteria, particularly rich on wood, were significantly different between artificial and natural substrates (SupplementaryFigure S6A, Supplementary Figure S7A and SupplementaryTable S8). Meanwhile, Planctomycetia, classified as Planctomycetes, was more abundant on artificial substrates (ANOVA, P-value < 0.001) (SupplementaryFigure S6A and SupplementaryTable S8).

For microeukaryotes, levels of Bacillariophyceae, Chlorophyceae, Ichthyosporea, Mediophyceae and Ulvophyceae were significantly higher on natural substrates (ANOVA, P-value < 0.05). However, the opposite trends were observed for the classes Clitellata and Ostracoda (ANOVA, P-value < 0.005) (SupplementaryTable S8). Additionally, compared with the other three substrates, the relative abundances of Clitellata, Trebouxiophyceae and Chytridiomycetes on wood were lowest (SupplementaryFigure S6B and Table S8).

In order to gain insights into the effect of substrate types on bacterial and microeukaryotic communities, the OTU abundance of bacteria and microeukaryotes between the artificial (ACF and PVC) and natural (pebble and wood) substrates was compared using STAMP. For bacteria, a total of 39 representative OTUs were screened and there were significant differences between natural and artificial substrates (SupplementaryFigure S8A and SupplementaryTable S9). Among the 39 OTUs, only four belong to the phyla Bacteroidetes and Proteobacteria, which were richer on natural substrates than on artificial substrates. Particularly, OTU127, assigned to Emticicia (bacteroidetes), had the biggest contribution to this change (SupplementaryFigure S8A). The other 35 OTUs, which appeared in excess on artificial substrates, were more diverse and mainly belonged to Planctomycetes (40%) and Proteobacteria (40%). Furthermore, OTU7, OTU32 and OTU44, which were annotated to Gemmata (Planctomycetes), Aquicella (Proteobacteria) and Bacillus (Firmicutes), respectively, were the three most diverse genera with the biggest difference between the artificial and natural substrates.

For microeukaryotes, a total of seven representative OTUs were screened and there were significant differences between natural and artificial substrates (q-value < 0.05) (SupplementaryFigure S8B and SupplementaryTable S9). In particular, OTU 2, belonging to Tubificoides (Annelida), contributes the most to the difference between natural and artificial substrates (SupplementaryFigure S8B). Interestingly, whether bacterial microeukaryotic communities, the OTUs shown in SupplementaryFigure S8 that were overrepresented on artificial substrates.

**Supplementary Table S1.** Physical and chemical parameters of water quality in the Xuanwu Lake (mean±SD).

| Parameter (unit) | Values |
| --- | --- |
| pH | 7.51 ± 0.32 |
| TN (mg/L) | 1.44 ± 0.12 |
| TP (mg/L) | 2.01 ± 0.30 |
| PO_4_^3-^ (mg/L) | 0.038 ± 0.007 |
| CO_3_^2-^ (mg/L) | 1.59 ± 0.16 |
| NO_3_^-^ (mg/L) | 0.68 ± 0.11 |
| NH_4_^+^(mg/L) | 0.46 ± 0.08 |

**Supplementary Table S2.** General characteristics of the substrates used in the experiments.

| Substrate type | Dimensions (mm)^a^ | Shape | Total surface area (mm^2^)^b^ |
| --- | --- | --- | --- |
| ACF | 100🞨50🞨1 | Schistose | 154500 |
| PVC | 100🞨50🞨1 | Schistose | 154500 |
| Pebble | Diameter 30 ± 5 | Axiolitic | 169560 |
| Wood | 100🞨50🞨5 | Schistose | 172500 |

^a^The substrate dimensions refer to the mean diameter for the spherical particles and average size for wood.

^b^The total surface area was calculated with 15 pieces of artificial substrates (ACF and PVC), and 600 particles of natural substrates (pebble and wood) loaded in the tanks.

**Supplementary Table S3.** Woods Hole culture medium (WC media) Recipe.

| Parameter | dosage |
| --- | --- |
| Biotin Vitamin solution (Sigma B-4639) | 0.0025g |
| Thiamine Vitamin Solution (Sigma T-1270) | 0.0335g |
| Vitamin B_12_ (Sigma V-6629) | 0.0135g |
| WC Trace Elements Solution | 50mL |
| NaNO_3_ (Fisher BP360-500) | 17.02g |
| CaCl_2_·2H_2_O (Sigma C-3881) | 7.352g |
| MgSO_4_·7H_2_O (Sigma 230391) | 7.394g |
| NaHCO_3_ (Fisher S 233) | 2.52g |
| Na_2_SiO_3_·9H_2_O (Sigma 307815) | 5.684g |
| K_2_HPO_4_ (Sigma P 3786) | 1.742g |
| H_3_BO_3_ (Baker 0084) | 4.8g |

**Supplementary Table S4.** Periphyton biomass (autotrophic and heterotrophic organisms) was evaluated by the determination of dry weight (DW) in three parallel samples under different cultivation time, and the results were expressed as g/m^2^, and the letters represent a significant difference at P-value < 0.05 by using one-way ANOVA.

| Cultivation time (day) | ACF | PVC | Pebble | Wood |
| --- | --- | --- | --- | --- |
| 3 | 1.05±0.04a | 1.03±0.12a | 0.96±0.10a | 1.27±0.29a |
| 7 | 2.06±0.32b | 2.87±0.20ab | 3.04±0.34ab | 3.65±0.31a |
| 10 | 8.12±0.18b | 8.07±0.10b | 5.75±0.48c | 9.46±0.55a |
| 14 | 16.36±0.46bc | 19.50±0.72ab | 15.41±1.17c | 22.11±1.39a |
| 21 | 33.76±0.99b | 42.91±3.02a | 27.54±2.68b | 41.69±1.34a |
| 28 | 53.09±1.52a | 60.67±2.16a | 35.41±2.55b | 60.67±3.27a |
| 35 | 57.35±3.30a | 55.63±5.29a | 33.85±1.39b | 57.36±2.28a |

**Supplementary Table S5.**Alpha diversity of bacteria and microeukaryotes, including Chao1, Observed species, Shannon, and Simpson indexes, for artificial (ACF and PVC) and natural (pebble and wood) substrates. And the letters represent a significant difference at p-value < 0.05 by using one-way ANOVA followed by Tukey's posthoc tests.

|  | Indexes | ACF | PVC | Pebble | Wood |
| --- | --- | --- | --- | --- | --- |
| Bacteria | Chao1 | 2682.784±54.955a | 2585.598±114.286ac | 2325.319±194.258b | 2461.358±91.976bc |
|  | Observed_  species | 1748±41a | 1662±87a | 1629±105a | 1657±33a |
|  | Shannon index | 7.963±0.081a | 7.957±0.226a | 7.940±0.086a | 7.713±0.144a |
|  | Simpson Index | 0.982±0.004ab | 0.987±0.002a | 0.987±0.002a | 0.979±0.003b |
| Eukaryotes | Chao1 | 426.059±45.631a | 428.649±55.880a | 428.325±27.197a | 431.495±22.580a |
|  | Observed_  species | 318±26a | 316±32a | 321±16a | 353±19a |
|  | Shannon index | 3.969±0.586b | 3.841±0.270b | 3.895±0.274b | 5.189±0.163a |
|  | Simpson Index | 0.858±0.062b | 0.849±0.033b | 0.850±0.040b | 0.936±0.008a |

**Supplementary Table S6.** The effects of substrate on the community variances assessed using permutational multivariate analyses of variance (PERMANOVA, ‘adonis and anosim’ in vegan R package) with 999 random permutations.

| Paired samples | | Adonis | | | Anosim | |
| --- | --- | --- | --- | --- | --- | --- |
|  |  | F | R^2^ | P | R | P |
| Bacteria | ACF-Pebble | 10.1 | 0.50 | 0.001 | 0.97 | 0.002 |
|  | ACF-PVC | 4.2 | 0.29 | 0.005 | 0.59 | 0.006 |
|  | ACF-Wood | 26.7 | 0.73 | 0.008 | 1.00 | 0.003 |
|  | Pebble-PVC | 3.7 | 0.27 | 0.004 | 0.63 | 0.003 |
|  | Pebble-Wood | 32.8 | 0.77 | 0.002 | 1.00 | 0.005 |
|  | PVC-Wood | 26.4 | 0.73 | 0.003 | 1.00 | 0.002 |
|  | ACF, PVC-Pebble,Wood | 4.9 | 0.18 | 0.006 | 0.30 | 0.003 |
| Microukaryotes | ACF-Pebble | 5.5 | 0.35 | 0.004 | 0.64 | 0.004 |
|  | ACF-PVC | 2.3 | 0.19 | 0.072 | 0.26 | 0.058 |
|  | ACF-Wood | 37.6 | 0.79 | 0.004 | 1.00 | 0.003 |
|  | Pebble-PVC | 4.5 | 0.31 | 0.003 | 0.54 | 0.003 |
|  | Pebble-Wood | 60.5 | 0.86 | 0.005 | 1.00 | 0.003 |
|  | PVC-Wood | 57.3 | 0.85 | 0.003 | 1.00 | 0.004 |
|  | ACF, PVC-Pebble,Wood | 8.42 | 0.28 | 0.002 | 0.41 | 0.001 |

**Supplementary Table S7.** The relative contribution of the most common phylum of bacteria and microeukaryotes to the biofilm communities from the four substrates: Activate carbon fiber (ACF), polyvinyl chloride (PVC), pebble and wood. Significant differences were conducted between artificial (ACF and PVC) and natural (pebble and wood) substrates by performing a one-way ANOVA followed by Tukey’s posthoc tests.

| Phylum (%) | | Artificial | | Natural | | P-values  (Artificial vs  Natural) |
| --- | --- | --- | --- | --- | --- | --- |
|  |  | ACF | PVC | Pebble | Wood |  |
| Bacteria | k__Bacteria;p__Acidobacteria | 2.87 | 2.82 | 2.35 | 2.51 | 0.13827 |
|  | k__Bacteria;p__Actinobacteria | 1.09 | 1.17 | 1.08 | 3.45 | 0.01676 |
|  | k__Bacteria;p__Bacteroidetes | 16.56 | 14.90 | 15.57 | 14.29 | 0.57485 |
|  | k__Bacteria;p__Chlorobi | 0.61 | 0.70 | 0.57 | 0.23 | 0.0094 |
|  | k__Bacteria;p__Chloroflexi | 1.09 | 1.50 | 1.65 | 2.17 | 0.00478 |
|  | k__Bacteria;p__Cyanobacteria | 9.99 | 18.44 | 22.67 | 8.01 | 0.69033 |
|  | k__Bacteria;p__Firmicutes | 8.49 | 5.24 | 7.00 | 5.07 | 0.30854 |
|  | k__Bacteria;p__Planctomycetes | 23.40 | 18.97 | 12.36 | 9.58 | 1.41963🞨10^-4^ |
|  | k__Bacteria;p__Proteobacteria | 28.93 | 31.25 | 30.76 | 49.28 | 0.00764 |
|  | k__Bacteria;p__Verrucomicrobia | 3.91 | 2.42 | 3.55 | 2.27 | 0.9192 |
|  | Others | 3.08 | 2.60 | 2.44 | 3.15 | 0.8047 |
| Microeukaryotes | k__Eukaryota;p__ | 24.34 | 21.53 | 20.48 | 49.22 | 0.03459 |
|  | k__Eukaryota;p__Bacillariophyta | 1.99 | 1.83 | 5.59 | 8.69 | 7.13862🞨10^-7^ |
|  | k__Eukaryota;p__Eustigmatophyceae | 0.25 | 0.32 | 1.14 | 4.70 | 3.06177🞨10^-4^ |
|  | k__Eukaryota;p__Intramacronucleata | 0.05 | 0.02 | 0.01 | 1.11 | 0.00667 |
|  | k__Fungi;p__Ascomycota | 0.45 | 0.80 | 0.69 | 1.79 | 0.00646 |
|  | k__Fungi;p__Basidiomycota | 0.58 | 0.30 | 0.23 | 1.59 | 0.04562 |
|  | k__Fungi;p__Chytridiomycota | 6.80 | 9.86 | 9.47 | 4.84 | 0.38764 |
|  | k__Metazoa;p__Annelida | 43.35 | 45.97 | 37.68 | 3.37 | 0.00191 |
|  | k__Metazoa;p__Arthropoda | 2.35 | 3.84 | 0.46 | 0.95 | 0.00271 |
|  | k__Metazoa;p__Gastrotricha | 3.64 | 2.06 | 0.34 | 0.01 | 4.02257🞨10^-7^ |
|  | k__Metazoa;p__Platyhelminthes | 9.28 | 3.71 | 12.24 | 2.48 | 0.71273 |
|  | k__Metazoa;p__Porifera | 0.00 | 0.01 | 0.01 | 1.01 | 0.00446 |
|  | k__Metazoa;p__Rotifera | 1.45 | 2.24 | 1.60 | 1.31 | 0.16127 |
|  | k__Viridiplantae;p__Chlorophyta | 4.94 | 6.46 | 9.22 | 17.62 | 5.4229🞨10^-5^ |
|  | Others | 0.53 | 1.05 | 0.85 | 1.30 | 0.06983 |

**Supplementary Table S8.** The relative contribution of the most common class of bacteria and microeukaryotes to the biofilm communities from the four substrates: Activate carbon fiber (ACF), polyvinyl chloride (PVC), pebble and wood. Significant differences were conducted between artificial (ACF and PVC) and natural (pebble and wood) substrates by performing a one-way ANOVA followed by Tukey’s posthoc tests.

| Class (%) | | Artificial | | Natural | | P-values  (Artificial vs Natural) |
| --- | --- | --- | --- | --- | --- | --- |
|  |  | ACF | PVC | Pebble | Wood |  |
| Bacteria | Acidimicrobiia | 0.44 | 0.53 | 0.45 | 1.36 | 0.02545 |
|  | Actinobacteria | 0.45 | 0.47 | 0.49 | 0.84 | 0.02762 |
|  | Alphaproteobacteria | 11.02 | 15.26 | 14.00 | 13.67 | 0.60096 |
|  | Anaerolineae | 0.73 | 1.14 | 1.33 | 1.65 | 0.0043 |
|  | Bacilli | 6.29 | 3.90 | 5.17 | 3.63 | 0.3509 |
|  | Bacteroidia | 0.19 | 0.16 | 0.19 | 0.82 | 0.00523 |
|  | Betaproteobacteria | 6.96 | 6.72 | 6.98 | 5.76 | 0.28583 |
|  | Clostridia | 2.18 | 1.32 | 1.82 | 1.27 | 0.28916 |
|  | Cytophagia | 3.00 | 1.79 | 2.13 | 1.89 | 0.28898 |
|  | Deltaproteobacteria | 1.84 | 1.68 | 1.44 | 4.35 | 0.02126 |
|  | Flavobacteriia | 0.44 | 0.53 | 0.53 | 2.68 | 0.00269 |
|  | Gammaproteobacteria | 8.81 | 7.28 | 7.93 | 23.97 | 0.00467 |
|  | Oscillatoriophycideae | 0.81 | 1.77 | 1.54 | 0.73 | 0.55147 |
|  | Phycisphaerae | 2.90 | 2.10 | 1.41 | 3.30 | 0.75805 |
|  | Planctomycetia | 20.18 | 16.69 | 10.76 | 7.18 | 6.2755🞨10^-5^ |
|  | Sphingobacteriia | 0.41 | 0.23 | 0.40 | 1.66 | 0.00167 |
|  | Synechococcophycideae | 6.02 | 14.36 | 18.85 | 7.51 | 0.25597 |
|  | Thermoleophilia | 0.20 | 0.16 | 0.14 | 1.04 | 0.01352 |
|  | Verrucomicrobiae | 2.72 | 1.96 | 2.91 | 2.01 | 0.8606 |
|  | [Chloracidobacteria] | 2.22 | 2.29 | 1.79 | 2.14 | 0.20034 |
|  | [Saprospirae] | 12.40 | 12.07 | 11.92 | 6.72 | 0.08125 |
|  | [Spartobacteria] | 0.64 | 0.25 | 0.31 | 0.23 | 0.0716 |
|  | Others | 9.15 | 7.33 | 7.50 | 5.59 | 0.03959 |
| Microeukaryotes | Agaricomycetes | 0.57 | 0.30 | 0.23 | 1.56 | 0.0468 |
|  | Bacillariophyceae | 1.63 | 1.35 | 3.74 | 5.20 | 3.40688🞨10^-4^ |
|  | Chlorophyceae | 1.18 | 2.13 | 3.16 | 14.44 | 4.59686🞨10^-4^ |
|  | Chytridiomycetes | 6.78 | 9.85 | 9.46 | 4.83 | 0.38717 |
|  | Clitellata | 43.35 | 45.97 | 37.68 | 3.37 | 0.00191 |
|  | Demospongiae | 0.00 | 0.01 | 0.01 | 1.01 | 0.00449 |
|  | Dothideomycetes | 0.27 | 0.13 | 0.05 | 1.04 | 0.04492 |
|  | Hexanauplia | 0.54 | 0.15 | 0.00 | 0.47 | 0.5986 |
|  | Ichthyosporea | 0.58 | 0.31 | 0.80 | 0.80 | 0.00401 |
|  | Labyrinthulomycetes | 0.28 | 0.15 | 0.16 | 0.96 | 0.017 |
|  | Mediophyceae | 0.33 | 0.45 | 1.78 | 3.26 | 3.2452🞨10^-7^ |
|  | Monogononta | 0.97 | 1.94 | 1.46 | 0.70 | 0.20568 |
|  | Ostracoda | 1.80 | 3.67 | 0.43 | 0.47 | 0.00213 |
|  | Trebouxiophyceae | 3.76 | 4.33 | 6.06 | 2.57 | 0.79358 |
|  | Ulvophyceae | 0.00 | 0.00 | 0.01 | 0.62 | 0.03359 |
|  | Others | 37.93 | 29.27 | 34.98 | 58.72 | 0.02177 |

**Supplementary Table S9.** Comparison of the bacterial and microeukaryotic operational taxonomic unit (OTU) abundance between the artificial (ACF and PVC) and natural (pebble and wood) substrates by STAMP. Significant differences were determined by Welch’s unequal variances t-test and then corrected for multiple tests according to the Benjamini–Hochberg false discovery rate (FDR) procedure. The q-values of bacteria were lower than 0.01, and those of microeukaryotes were lower than 0.05.

| Category | OTU ID | Artificial: mean rel. freq. (%) | Artificial: std. dev. (%) | Natural: mean rel. freq. (%) | Natural: std. dev. (%) | p-values | p-values (corrected) | Difference between means | 95.0% lower CI | 95.0% upper CI | Taxonomy |
| --- | --- | --- | --- | --- | --- | --- | --- | --- | --- | --- | --- |
| Bacteria | OTU102 | 0.287105802 | 0.055891402 | 0.079698709 | 0.040840418 | 3.28E-09 | 9.46E-06 | 0.207407093 | 0.163889516 | 0.25092467 | k__Bacteria; p__Proteobacteria; c__Betaproteobacteria; o__;  f__;  g__;  s__ |
|  | OTU127 | 0.048515808 | 0.032887521 | 0.200671391 | 0.058434093 | 7.34E-07 | 0.00052942 | -0.152155583 | -0.194747976 | -0.109563191 | k__Bacteria; p__Bacteroidetes; c__Cytophagia; o__Cytophagales; f__Cytophagaceae; g__Emticicia;  s__ |
|  | OTU138 | 0.190138956 | 0.045928504 | 0.056588592 | 0.047305683 | 9.46E-07 | 0.000545908 | 0.133550364 | 0.092320172 | 0.174780556 | k__Bacteria; p__Planctomycetes; c__Planctomycetia; o__Pirellulales; f__Pirellulaceae;  g__;  s__ |
|  | OTU1534 | 0.00594788 | 0.002096622 | 0.000815963 | 0.001159464 | 1.68E-06 | 0.000691906 | 0.005131918 | 0.00360886 | 0.006654976 | k__Bacteria; p__Proteobacteria; c__Gammaproteobacteria;  o__Legionellales; f__Coxiellaceae; g__Aquicella;  s__ |
|  | OTU165 | 0.071964694 | 0.023291841 | 0.128135349 | 0.027204701 | 3.47E-05 | 0.006679565 | -0.056170655 | -0.078595545 | -0.033745764 | k__Bacteria; p__Bacteroidetes; c__[Saprospirae]; o__[Saprospirales]; f__Chitinophagaceae; g__;  s__ |
|  | OTU170 | 0.148524168 | 0.048597207 | 0.040443257 | 0.022699074 | 6.02E-06 | 0.001828074 | 0.108080911 | 0.073722441 | 0.142439382 | k__Bacteria; p__Planctomycetes; c__Planctomycetia; o__Pirellulales; f__Pirellulaceae; g__A17;  s__ |
|  | OTU183 | 0.119595857 | 0.036531638 | 0.026045476 | 0.025408098 | 1.01E-06 | 0.000530445 | 0.093550382 | 0.065528906 | 0.121571857 | k__Bacteria; p__Proteobacteria; c__Gammaproteobacteria;  o__Legionellales; f__Coxiellaceae;  g__;  s__ |
|  | OTU190 | 0.106349454 | 0.040238011 | 0.030233859 | 0.015355656 | 3.98E-05 | 0.007396167 | 0.076115595 | 0.048289556 | 0.103941635 | k__Bacteria; p__Proteobacteria; c__Gammaproteobacteria;  o__Legionellales |
|  | OTU196 | 0.125266145 | 0.021257892 | 0.024667455 | 0.022315896 | 2.87E-10 | 1.66E-06 | 0.100598691 | 0.081324176 | 0.119873205 | k__Bacteria; p__Proteobacteria; c__Betaproteobacteria; o__;  f__;  g__;  s__ |
|  | OTU199 | 0.10158582 | 0.020562076 | 0.034511637 | 0.034329165 | 2.82E-05 | 0.005804614 | 0.067074183 | 0.04172516 | 0.092423206 | k__Bacteria; p__Proteobacteria; c__Alphaproteobacteria; o__Rhizobiales; f__Rhizobiaceae |
|  | OTU2013 | 0.028958632 | 0.008478621 | 0.005318535 | 0.007552039 | 6.67E-07 | 0.000549198 | 0.023640097 | 0.016534822 | 0.030745371 | k__Bacteria; p__Planctomycetes; c__Planctomycetia; o__Gemmatales; f__Gemmataceae; g__Gemmata;  s__ |
|  | OTU216 | 0.097631429 | 0.018333013 | 0.035877239 | 0.017613173 | 5.31E-08 | 0.000102179 | 0.061754189 | 0.045855875 | 0.077652504 | k__Bacteria; p__Proteobacteria; c__Gammaproteobacteria;  o__Legionellales; f__Coxiellaceae; g__Aquicella;  s__ |
|  | OTU2388 | 0.031432679 | 0.012663609 | 0.00588732 | 0.005457914 | 1.91E-05 | 0.004580129 | 0.025545359 | 0.016680755 | 0.034409963 | k__Bacteria;  p__TM6;  c__SJA-4;  o__S1198;  f__;  g__;  s__ |
|  | OTU2501 | 0.029340101 | 0.008541841 | 0.006305396 | 0.005702998 | 4.58E-07 | 0.000527854 | 0.023034704 | 0.01655731 | 0.029512098 | k__Bacteria; p__Bacteroidetes; c__[Saprospirae]; o__[Saprospirales]; f__Saprospiraceae; g__;  s__ |
|  | OTU261 | 0.070899511 | 0.030396196 | 0.014391764 | 0.015551712 | 4.54E-05 | 0.007943658 | 0.056507747 | 0.03472609 | 0.078289403 | k__Bacteria |
|  | OTU294 | 0.063218406 | 0.018728025 | 0.020786046 | 0.014467314 | 7.10E-06 | 0.002047255 | 0.04243236 | 0.027579682 | 0.057285038 | k__Bacteria; p__Planctomycetes; c__Planctomycetia; o__Pirellulales; f__Pirellulaceae;  g__;  s__ |
|  | OTU32 | 0.835543922 | 0.210755484 | 0.199477097 | 0.19187454 | 2.21E-07 | 0.000318657 | 0.636066825 | 0.457757297 | 0.814376353 | k__Bacteria; p__Proteobacteria; c__Gammaproteobacteria;  o__Legionellales; f__Coxiellaceae; g__Aquicella;  s__ |
|  | OTU320 | 0.051034303 | 0.021481625 | 0.011668412 | 0.00991472 | 5.26E-05 | 0.00842917 | 0.039365892 | 0.024202318 | 0.054529466 | k__Bacteria;  p__TM6;  c__SJA-4;  o__;  f__;  g__;  s__ |
|  | OTU326 | 0.054569423 | 0.013823168 | 0.016829289 | 0.013063518 | 1.30E-06 | 0.000626151 | 0.037740134 | 0.02584521 | 0.049635058 | k__Bacteria; p__Planctomycetes; c__Planctomycetia; o__Pirellulales; f__Pirellulaceae;  g__;  s__ |
|  | OTU338 | 0.046984006 | 0.013864085 | 0.013980497 | 0.010595774 | 3.51E-06 | 0.001189955 | 0.033003509 | 0.02204866 | 0.043958358 | k__Bacteria; p__Proteobacteria; c__Deltaproteobacteria;  o__FAC87;  f__;  g__;  s__ |
|  | OTU3810 | 0.031001309 | 0.008110233 | 0.013262885 | 0.007085744 | 1.84E-05 | 0.004612019 | 0.017738424 | 0.010997211 | 0.024479638 | k__Bacteria; p__Proteobacteria; c__Betaproteobacteria; o__Burkholderiales; f__Comamonadaceae;  g__;  s__ |
|  | OTU385 | 0.042388984 | 0.012716068 | 0.008907693 | 0.005426438 | 8.67E-07 | 0.000555575 | 0.033481291 | 0.024589879 | 0.042372702 | k__Bacteria; p__Planctomycetes; c__Planctomycetia; o__B97;  f__;  g__;  s__ |
|  | OTU3970 | 0.049003826 | 0.013877698 | 0.022284212 | 0.011245153 | 6.49E-05 | 0.009846847 | 0.026719614 | 0.015522803 | 0.037916425 | k__Bacteria; p__Proteobacteria; c__Alphaproteobacteria;  o__Rhizobiales;  f__;  g__;  s__ |
|  | OTU407 | 0.038143276 | 0.01043158 | 0.010521319 | 0.007161897 | 6.14E-07 | 0.000590681 | 0.027621957 | 0.019650143 | 0.03559377 | k__Bacteria; p__Proteobacteria; c__Alphaproteobacteria;  o__Rickettsiales;  f__;  g__;  s__ |
|  | OTU421 | 0.058718201 | 0.018831031 | 0.014591218 | 0.012837633 | 3.35E-06 | 0.001208294 | 0.044126983 | 0.029764949 | 0.058489018 | k__Bacteria; p__Planctomycetes; c__Planctomycetia; o__Gemmatales; f__Gemmataceae; g__Gemmata;  s__ |
|  | OTU44 | 0.886762196 | 0.329763583 | 0.263691522 | 0.129362035 | 3.98E-05 | 0.007170504 | 0.623070673 | 0.394459048 | 0.851682298 | k__Bacteria; p__Firmicutes; c__Bacilli; o__Bacillales; f__Bacillaceae; g__Bacillus; s__selenatarsenatis |
|  | OTU4495 | 0.057146502 | 0.020131402 | 0.020251292 | 0.00928813 | 5.26E-05 | 0.008661123 | 0.03689521 | 0.022685421 | 0.051104999 | k__Bacteria; p__Firmicutes; c__Bacilli; o__Bacillales; f__Planococcaceae; g__Sporosarcina; s__ginsengi |
|  | OTU540 | 0.006118033 | 0.003561526 | 0.028196673 | 0.009601277 | 4.99E-06 | 0.001598461 | -0.02207864 | -0.028702271 | -0.015455009 | k__Bacteria; p__Proteobacteria; c__Gammaproteobacteria; o__Xanthomonadales; f__Sinobacteraceae; g__;  s__ |
|  | OTU544 | 0.024122679 | 0.008130004 | 0.004124787 | 0.004925023 | 1.56E-06 | 0.000694314 | 0.019997892 | 0.013979417 | 0.026016368 | k__Bacteria; p__Planctomycetes; c__Planctomycetia; o__Pirellulales; f__Pirellulaceae;  g__;  s__ |
|  | OTU55 | 0.411961954 | 0.108689901 | 0.118388905 | 0.109207622 | 2.33E-06 | 0.000895293 | 0.29357305 | 0.197228995 | 0.389917104 | k__Bacteria; p__Planctomycetes; c__Planctomycetia; o__Pirellulales; f__Pirellulaceae;  g__;  s__ |
|  | OTU6028 | 0.572746437 | 0.20954482 | 0.132641874 | 0.138871283 | 1.33E-05 | 0.003485733 | 0.440104563 | 0.281519328 | 0.598689798 | k__Bacteria; p__Planctomycetes; c__Planctomycetia; o__Gemmatales; f__Gemmataceae; g__Gemmata;  s__ |
|  | OTU6894 | 0.026363747 | 0.010073739 | 0.005141165 | 0.00524313 | 1.10E-05 | 0.003013447 | 0.021222583 | 0.013983404 | 0.028461761 | k__Bacteria; p__Planctomycetes; c__Planctomycetia; o__Gemmatales; f__Gemmataceae; g__Gemmata;  s__ |
|  | OTU69 | 0.319060567 | 0.097752238 | 0.124991959 | 0.04536694 | 2.21E-05 | 0.005101017 | 0.194068608 | 0.125016404 | 0.263120812 | k__Bacteria; p__Proteobacteria; c__Gammaproteobacteria;  o__Legionellales  f__;  g__;  s__ |
|  | OTU7 | 3.177463742 | 0.887833117 | 1.011639288 | 1.01426696 | 2.52E-05 | 0.005384376 | 2.165824454 | 1.32209575 | 3.009553158 | k__Bacteria; p__Planctomycetes; c__Planctomycetia; o__Gemmatales; f__Gemmataceae; g__Gemmata;  s__ |
|  | OTU714 | 0.003851023 | 0.003645873 | 0.015736626 | 0.006397104 | 4.81E-05 | 0.008157552 | -0.011885603 | -0.016560059 | -0.007211147 | k__Bacteria; p__Proteobacteria; c__Deltaproteobacteria; o__Bdellovibrionales; f__Bdellovibrionaceae;  g__Bdellovibrio;  s__ |
|  | OTU73 | 0.574781033 | 0.263117643 | 0.093489851 | 0.078397455 | 6.15E-05 | 0.00958508 | 0.481291181 | 0.302369377 | 0.660212986 | k__Bacteria; p__Bacteroidetes; c__Cytophagia; o__Cytophagales; f__Cytophagaceae |
|  | OTU8713 | 0.006329003 | 0.003244296 | 0.000451778 | 0.001063144 | 6.51E-05 | 0.009623449 | 0.005877225 | 0.003659071 | 0.008095379 | k__Bacteria; p__Planctomycetes; c__Planctomycetia; o__Gemmatales; f__Gemmataceae; g__Gemmata;  s__ |
|  | OTU9004 | 0.02287964 | 0.009996699 | 0.002797237 | 0.003046714 | 2.42E-05 | 0.00537142 | 0.020082403 | 0.013276474 | 0.026888332 | k__Bacteria; p__Proteobacteria; c__Gammaproteobacteria;  o__Legionellales; f__Coxiellaceae; g__Aquicella;  s__ |
|  | OTU9772 | 0.088734672 | 0.038862197 | 0.013326118 | 0.016249925 | 2.94E-05 | 0.00583818 | 0.075408554 | 0.048295191 | 0.102521917 | k__Bacteria; p__Planctomycetes; c__Planctomycetia; o__Gemmatales; f__Gemmataceae; g__Gemmata;  s__ |
| Microeukaryotes | OTU10 | 2.616333972 | 1.176572202 | 0.138392121 | 0.156691571 | 2.09E-05 | 0.023814528 | 2.477941851 | 1.693527707 | 3.262355995 | k__Metazoa; p__Gastrotricha;  c__;  o__Chaetonotida; f__Chaetonotidae;  g__;  s__ |
|  | OTU1396 | 0.036159088 | 0.014835404 | 0.009200614 | 0.010199781 | 7.97E-05 | 0.015101681 | 0.026958474 | 0.015616706 | 0.038300242 | k__Metazoa; p__Annelida; c__Clitellata; o__Haplotaxida; f__Tubificidae; g__Tubificoides；  s__ |
|  | OTU1674 | 0.005413114 | 0.002125192 | 0.001171335 | 0.001644024 | 3.60E-05 | 0.010235438 | 0.004241779 | 0.002555521 | 0.005928036 | k__Metazoa; p__Annelida; c__Clitellata; o__Haplotaxida; f__Tubificidae; g__Tubificoides；  s__ |
|  | OTU2 | 20.97849289 | 7.70585981 | 5.672660083 | 4.049301178 | 2.18E-05 | 0.012385128 | 15.30583281 | 9.759266301 | 20.85239932 | k__Metazoa; p__Annelida; c__Clitellata; o__Haplotaxida; f__Tubificidae; g__Tubificoides；  s__ |
|  | OTU23 | 0.458192531 | 0.235016693 | 1.774812791 | 0.764359349 | 0.000107407 | 0.017445926 | -1.31662026 | -1.837259484 | -0.795981036 | k__Viridiplantae; p__Chlorophyta; c__Chlorophyceae; o__Sphaeropleales; f__Scenedesmaceae; g__Scenedesmus；  s__ |
|  | OTU29 | 0.082857376 | 0.030223011 | 0.985922341 | 0.432084169 | 2.41E-05 | 0.009146599 | -0.903064965 | -1.190166655 | -0.615963275 | k__Eukaryota; p__Bacillariophyta; c__Mediophyceae; o__;  f__;  g__；  s__ |
|  | OTU879 | 0.020256164 | 0.008259581 | 0.00469435 | 0.005078579 | 4.41E-05 | 0.010029809 | 0.015561814 | 0.00942653 | 0.021697098 | k__Metazoa; p__Annelida; c__Clitellata; o__Haplotaxida; f__Tubificidae; g__Limnodriloides；  s__ |

**Supplementary Table S10.** The proportions of nodes of the most common phylum of bacteria and microeukaryotes in the network for the four substrates: ACF, PVC, pebble and wood. Significant differences were conducted between artificial (ACF and PVC) and natural (pebble and wood) substrates by performing a one-way ANOVA followed by Tukey’s posthoc tests.

| Phylum (%) | | Artificial | | Natural | |
| --- | --- | --- | --- | --- | --- |
|  |  | ACF | PVC | Pebble | Wood |
| Bacteria | Acidobacteria | 3.47 | 3.11 | 2.86 | 2.79 |
|  | Actinobacteria | 2.56 | 2.92 | 2.29 | 4.66 |
|  | Bacteroidetes | 10.24 | 12.84 | 14.5 | 19.37 |
|  | Chloroflexi | 2.56 | 3.11 | 3.05 | 3.17 |
|  | Cyanobacteria | 6.58 | 8.17 | 10.11 | 5.76 |
|  | Firmicutes | 7.13 | 6.23 | 7.06 | 4.47 |
|  | Planctomycetes | 21.39 | 19.07 | 16.79 | 15.64 |
|  | Proteobacteria | 34.37 | 35.41 | 33.78 | 34.26 |
|  | Verrucomicrobia | 4.57 | 2.92 | 2.86 | 3.35 |
|  | Others | 7.13 | 6.22 | 6.70 | 6.53 |
| Microeukaryotes | Annelida | 8.62 | 7.21 | 7.34 | 2.47 |
|  | Arthropoda | 4.31 | 3.60 | 2.75 | 1.85 |
|  | Ascomycota | 6.90 | 5.41 | 6.42 | 6.79 |
|  | Bacillariophyta | 5.17 | 9.01 | 12.84 | 8.64 |
|  | Chlorophyta | 10.34 | 15.32 | 17.43 | 15.43 |
|  | Chytridiomycota | 2.59 | 6.31 | 3.67 | 7.41 |
|  | Gastrotricha | 3.45 | 2.70 | 1.83 | 0.00 |
|  | Platyhelminthes | 5.17 | 1.80 | 1.83 | 2.47 |
|  | Rotifera | 8.62 | 12.61 | 8.26 | 4.32 |
|  | Zoopagomycota | 0.86 | 1.80 | 0.92 | 0.62 |
|  | Others | 43.97 | 34.23 | 36.71 | 50.00 |

**Supplementary Table S11.** Key topological features of bacterial and microeukaryotic networks of periphytic biofilms colonized on artificial (ACF and PVC) and natural (pebble and wood) substrates.

| Network Indexes | Bacteria | | | | Microeukaryotes | | | |
| --- | --- | --- | --- | --- | --- | --- | --- | --- |
|  | ACF | PVC | Pebble | Wood | ACF | PVC | Pebble | Wood |
| Number of nodes | 547 | 514 | 524 | 537 | 116 | 111 | 109 | 162 |
| Number of edges | 5306 | 4622 | 2890 | 3129 | 514 | 286 | 385 | 773 |
| Positive (%) | 74.5 | 66.79 | 63.49 | 62.35 | 82.88 | 83.92 | 85.19 | 66.75 |
| Negative (%) | 25.5 | 33.21 | 36.51 | 37.65 | 17.12 | 16.08 | 14.81 | 33.25 |
| Average clustering coefficient | 0.505 | 0.483 | 0.452 | 0.418 | 0.637 | 0.529 | 0.514 | 0.523 |
| Average degree | 19.4 | 17.984 | 11.031 | 11.654 | 8.862 | 5.153 | 7.064 | 9.543 |
| Average path length | 3.958 | 3.974 | 4.474 | 4.491 | 3.535 | 5.06 | 5.123 | 4.115 |
| Graph density | 0.036 | 0.035 | 0.021 | 0.022 | 0.077 | 0.047 | 0.065 | 0.059 |
| Modularity | 0.653 | 0.558 | 0.647 | 0.647 | 0.55 | 0.671 | 0.494 | 0.62 |
| Network diameter | 13 | 12 | 10 | 13 | 10 | 14 | 16 | 12 |

**Supplementary Table S12.** The keystone species of bacteria and microeukaryotes in the network for the four substrates: Activate carbon fiber (ACF), polyvinyl chloride (PVC), pebble and wood. Significant differences were conducted between artificial (ACF and PVC) and natural (pebble and wood) substrates.

| Category | Substrate | Role | OTU ID | Taxonomy |
| --- | --- | --- | --- | --- |
| Bacteria | ACF | Connector | OTU120 | k__Bacteria;  p__Bacteroidetes；  c__Sphingobacteriia； o__Sphingobacteriales；  f__；  g__；  s__ |
| Bacteria | ACF | Connector | OTU165 | k__Bacteria；  p__Bacteroidetes；  c__[Saprospirae]；  o__[Saprospirales]； f__Chitinophagaceae；  g__；  s__ |
| Bacteria | ACF | Connector | OTU170 | k__Bacteria；  p__Planctomycetes；  c__Planctomycetia；  o__Pirellulales；  f__Pirellulaceae  g__A17；  s__ |
| Bacteria | ACF | Connector | OTU338 | k__Bacteria；  p__Proteobacteria； c__Deltaproteobacteria；  o__FAC87；  f__；  g__；  s__ |
| Bacteria | ACF | Connector | OTU404 | k__Bacteria；  p__Actinobacteria；  c__Acidimicrobiia；  o__Acidimicrobiales；  f__C111；  g__；  s__ |
| Bacteria | ACF | Connector | OTU4786 | k__Bacteria；  p__Proteobacteria；  c__Betaproteobacteria；  o__Ellin6067；  f__；  g__；  s__ |
| Bacteria | ACF | Connector | OTU763 | k__Bacteria；  p__Chlamydiae；  c__Chlamydiia；  o__Chlamydiales；  f__；  g__；  s__ |
| Bacteria | ACF | Connector | OTU588 | k__Bacteria；  p__Planctomycetes；  c__Planctomycetia；  o__Pirellulales；  f__Pirellulaceae；  g__；  s__ |
| Bacteria | ACF | Module hub | OTU331 | k__Bacteria；  p__Planctomycetes；  c__Planctomycetia；  o__Gemmatales；  f__Gemmataceae；  g__Gemmata；  s__ |
| Bacteria | PVC | Connector | OTU1229 | k__Bacteria；  p__Proteobacteria；  c__Betaproteobacteria；  o__Burkholderiales |
| Bacteria | PVC | Connector | OTU216 | k__Bacteria；  p__Proteobacteria；  c__Gammaproteobacteria；  o__Legionellales；  f__Coxiellaceae；  g__Aquicella；  s__ |
| Bacteria | PVC | Connector | OTU305 | k__Bacteria；  p__Actinobacteria  c__Acidimicrobiia；  o__Acidimicrobiales；  f__；  g__；  s__ |
| Bacteria | PVC | Connector | OTU319 | k__Bacteria；  p__Proteobacteria；  c__Alphaproteobacteria；  o__Rhodospirillales； f__Acetobacteraceae；  g__；  s__ |
| Bacteria | PVC | Connector | OTU4895 | k__Bacteria；  p__Firmicutes；  c__Bacilli；  o__Bacillales；  f__Bacillaceae；  g__Bacillus；  s__flexus |
| Bacteria | PVC | Module hub | OTU249 | k__Bacteria；  p__Bacteroidetes；  c__[Saprospirae]；  o__[Saprospirales]；f__Chitinophagaceae； g__Sediminibacterium；  s__ |
| Bacteria | PVC | Module hub | OTU3724 | k__Bacteria；  p__Planctomycetes；  c__Planctomycetia； o__Planctomycetales； f__Planctomycetaceae； g__Planctomyces；  s__ |
| Bacteria | PVC | Module hub | OTU43 | k__Bacteria；  p__Acidobacteria； c__[Chloracidobacteria]；  o__RB41；  f__Ellin6075；  g__；  s__ |
| Bacteria | Pebble | Connector | OTU107 | k__Bacteria；  p__Planctomycetes；  c__Planctomycetia；  o__Gemmatales；  f__Gemmataceae；  g__Gemmata；  s__ |
| Bacteria | Pebble | Connector | OTU120 | k__Bacteria；  p__Bacteroidetes；  c__Sphingobacteriia；  o__Sphingobacteriales；  f__；  g__；  s__ |
| Bacteria | Pebble | Connector | OTU164 | k__Bacteria；  p__Proteobacteria； c__Alphaproteobacteria； o__Sphingomonadales； f__Sphingomonadaceae； g__Novosphingobium；  s__ |
| Bacteria | Pebble | Connector | OTU201 | k__Bacteria；  p__Cyanobacteria；c__Oscillatoriophycideae； o__Chroococcales；  f__；  g__；  s__ |
| Bacteria | Pebble | Connector | OTU206 | k__Bacteria；  p__Planctomycetes；  c__Planctomycetia；  o__Pirellulales；  f__Pirellulaceae；  g__；  s__ |
| Bacteria | Pebble | Connector | OTU213 | k__Bacteria；  p__Planctomycetes；  c__Planctomycetia；  o__Gemmatales；  f__Gemmataceae；  g__；  s__ |
| Bacteria | Pebble | Connector | OTU23 | k__Bacteria；  p__Cyanobacteria；  c__Synechococcophycideae； o__Synechococcales；f__Acaryochloridaceae；g__Acaryochloris；  s__ |
| Bacteria | Pebble | Connector | OTU2603 | k__Bacteria；  p__Firmicutes；  c__Bacilli；  o__Bacillales；  f__Bacillaceae；  g__Bacillus |
| Bacteria | Pebble | Connector | OTU2672 | k__Bacteria；  p__Firmicutes；  c__Bacilli；  o__Bacillales；  f__Bacillacea；  g__Bacillus；  s__ |
| Bacteria | Pebble | Connector | OTU299 | k__Bacteria；  p__Proteobacteria； c__Betaproteobacteria； o__Burkholderiales； f__Comamonadaceae； g__Hydrogenophaga；  s__ |
| Bacteria | Pebble | Connector | OTU3139 | k__Bacteria；  p__Firmicutes；  c__Bacilli；  o__Bacillales；  f__Bacillaceae；  g__Bacillus；  s__ginsengihumi |
| Bacteria | Pebble | Connector | OTU35 | k__Bacteria；  p__Planctomycetes；  c__Planctomycetia；  o__Gemmatales；  f__Gemmataceae；  g__Gemmata s__ |
| Bacteria | Pebble | Connector | OTU352 | k__Bacteria；  p__Cyanobacteria；  c__Synechococcophycideae； o__Synechococcales； f__Acaryochloridaceae；  g__；  s__ |
| Bacteria | Pebble | Connector | OTU356 | k__Bacteria；  p__Acidobacteria；  c__Solibacteres；  o__Solibacterales；f__[Bryobacteraceae] |
| Bacteria | Pebble | Connector | OTU370 | k__Bacteria；  p__Proteobacteria； c__Alphaproteobacteria；  o__Rhizobiales；  f__Hyphomicrobiaceae； g__Pedomicrobium；  s__ |
| Bacteria | Pebble | Connector | OTU3970 | k__Bacteria；  p__Proteobacteria； c__Alphaproteobacteria；  o__Rhizobiales；  f__；  g__；  s__ |
| Bacteria | Pebble | Connector | OTU40 | k__Bacteria；  p__Cyanobacteria； c__Synechococcophycideae； o__Pseudanabaenales； f__Pseudanabaenaceae ；g__Leptolyngbya；  s__ |
| Bacteria | Pebble | Connector | OTU434 | k__Bacteria；  p__Cyanobacteria |
| Bacteria | Pebble | Connector | OTU442 | k__Bacteria；  p__Proteobacteria； c__Alphaproteobacteria ；o__Rhodospirillales； f__Acetobacteraceae；  g__；  s__ |
| Bacteria | Pebble | Connector | OTU4760 | k__Bacteria；  p__Proteobacteria；c__Gammaproteobacteria； o__Xanthomonadales； f__Xanthomonadaceae；  g__；  s__ |
| Bacteria | Pebble | Connector | OTU6665 | k__Bacteria；  p__Proteobacteria； c__Betaproteobacteria； o__Burkholderiales； f__Burkholderiaceae；  g__；  s__ |
| Bacteria | Pebble | Connector | OTU687 | k__Bacteria；  p__Firmicutes；  c__Clostridia；  o__Clostridiales；  f__Lachnospiraceae；  g__Defluviitalea；  s__saccharophila |
| Bacteria | Pebble | Connector | OTU57 | k__Bacteria；  p__Planctomycetes；  c__Planctomycetia； o__Planctomycetales； f__Planctomycetaceae； g__Planctomyces；  s__ |
| Bacteria | Pebble | Connector | OTU650 | k__Bacteria；  p__Proteobacteria； c__Alphaproteobacteria； o__Rhodospirillales； f__Acetobacteraceae；  g__；  s__ |
| Bacteria | Pebble | Connector | OTU70 | k__Bacteria；  p__Cyanobacteria； c__Synechococcophycideae； o__Pseudanabaenales； f__Pseudanabaenaceae； g__Leptolyngbya；  s__ |
| Bacteria | Pebble | Connector | OTU66 | k__Bacteria；  p__Planctomycetes；  c__Planctomycetia；  o__Gemmatales；  f__Gemmataceae；  g__；  s__ |
| Bacteria | Pebble | Module hub | OTU102 | k__Bacteria；  p__Proteobacteria； c__Betaproteobacteria；  o__；  f__；  g__；  s__ |
| Bacteria | Pebble | Module hub | OTU1820 | k__Bacteria；  p__Proteobacteria； c__Betaproteobacteria； o__Hydrogenophilales； f__Hydrogenophilaceae； g__Thiobacillus；  s__ |
| Bacteria | Pebble | Module hub | OTU215 | k__Bacteria；  p__Firmicutes；  c__Clostridia；  o__OPB54；  f__；  g__；  s__ |
| Bacteria | Pebble | Module hub | OTU3 | k__Bacteria；  p__Proteobacteria； c__Alphaproteobacteria； o__Sphingomonadales； f__Sphingomonadaceae； g__Sandaracinobacter；  s__sibiricus |
| Bacteria | Wood | Connector | OTU1149 | k__Bacteria；  p__Chloroflexi；  c__Anaerolineae;  o__SBR1031;  f__A4b;  g__;  s__ |
| Bacteria | Wood | Connector | OTU147 | k__Bacteria;  p__Planctomycetes;  c__Planctomycetia;  o__Gemmatales;  f__Gemmataceae;  g__Gemmata;  s__ |
| Bacteria | Wood | Connector | OTU263 | k__Bacteria;  p__Proteobacteria; c__Gammaproteobacteria; o__Xanthomonadales; f__Xanthomonadaceae;  g__;  s__ |
| Bacteria | Wood | Connector | OTU276 | k__Bacteria;  p__Planctomycetes;  c__Planctomycetia;  o__Pirellulales;  f__Pirellulaceae;  g__;  s__ |
| Bacteria | Wood | Connector | OTU300 | k__Bacteria;  p__Proteobacteria; c__Alphaproteobacteria; o__Rhodospirillales; f__Rhodospirillaceae;  g__;  s__ |
| Bacteria | Wood | Connector | OTU32 | k__Bacteria;  p__Proteobacteria; c__Gammaproteobacteria; o__Legionellales;  f__Coxiellaceae;  g__Aquicella;  s__ |
| Bacteria | Wood | Connector | OTU334 | k__Bacteria;  p__Proteobacteria; c__Alphaproteobacteria;  o__  f__;  g__;  s__ |
| Bacteria | Wood | Connector | OTU35 | k__Bacteria;  p__Planctomycetes;  c__Planctomycetia;  o__Gemmatales;  f__Gemmataceae;  g__Gemmata;  s__ |
| Bacteria | Wood | Connector | OTU400 | k__Bacteria;  p__Proteobacteria; c__Alphaproteobacteria; o__Sphingomonadales; f__Erythrobacteraceae;  g__;  s__ |
| Bacteria | Wood | Connector | OTU404 | k__Bacteria;  p__Actinobacteria;  c__Acidimicrobiia;  o__Acidimicrobiales;  f__C111;  g__;  s__ |
| Bacteria | Wood | Connector | OTU441 | k__Bacteria;  p__Acidobacteria;  c__Solibacteres;  o__Solibacterales;  f__;  g__;  s__ |
| Bacteria | Wood | Connector | OTU4676 | k__Bacteria;  p__Acidobacteria;  c__[Chloracidobacteria];  o__PK29;  f__;  g__;  s__ |
| Bacteria | Wood | Connector | OTU468 | k__Bacteria;  p__Proteobacteria; c__Deltaproteobacteria; o__Syntrophobacterales; f__Syntrophobacteraceae;  g__;  s__ |
| Bacteria | Wood | Connector | OTU478 | k__Bacteria;  p__Acidobacteria;  c__Solibacteres;  o__Solibacterales;  f__;  g__;  s__ |
| Bacteria | Wood | Connector | OTU480 | k__Bacteria;  p__Actinobacteria;  c__Actinobacteria;  o__Actinomycetales;  f__Microbacteriaceae;  g__Cryocola;  s__ |
| Bacteria | Wood | Connector | OTU502 | k__Bacteria;  p__Proteobacteria; c__Deltaproteobacteria; o__Bdellovibrionales; f__Bdellovibrionaceae;  g__Bdellovibrio;  s__ |
| Bacteria | Wood | Connector | OTU533 | k__Bacteria;  p__Planctomycetes;  c__Planctomycetia;  o__Pirellulales;  f__Pirellulaceae;  g__Pirellula;  s__ |
| Bacteria | Wood | Connector | OTU616 | k__Bacteria;  p__Bacteroidetes;  c__Sphingobacteriia; o__Sphingobacteriales;  f__;  g__;  s__ |
| Bacteria | Wood | Connector | OTU64 | k__Bacteria;  p__Proteobacteria; c__Gammaproteobacteria; o__Thiotrichales;  f__Piscirickettsiaceae;  g__;  s__ |
| Bacteria | Wood | Connector | OTU688 | k__Bacteria;  p__Planctomycetes;  c__Planctomycetia  o__Gemmatales;  f__Gemmataceae;  g__;  s__ |
| Bacteria | Wood | Connector | OTU863 | k__Bacteria;  p__Bacteroidetes;  c__Sphingobacteriia; o__Sphingobacteriales  f__  g__  s__ |
| Bacteria | Wood | Connector | OTU749 | k__Bacteria;  p__Proteobacteria; c__Alphaproteobacteria;  o__BD7-3 ;  f__ ;  g__;  s__ |
| Bacteria | Wood | Connector | OTU706 | k__Bacteria;  p__Bacteroidetes;  c__Cytophagia;  o__Cytophagales |
| Bacteria | Wood | Connector | OTU779 | k__Bacteria;  p__Proteobacteria; c__Deltaproteobacteria; o__Bdellovibrionales; f__Bdellovibrionaceae;  g__Bdellovibrio;  s__ |
| Bacteria | Wood | Module hub | OTU11 | k__Bacteria;  p__Cyanobacteria;  c__;  o__;  f__;  g__;  s__ |
| Bacteria | Wood | Module hub | OTU256 | k__Bacteria;  p__Bacteroidetes;  c__[Saprospirae];  o__[Saprospirales]; f__Chitinophagaceae;  g__;  s__ |
| Bacteria | Wood | Module hub | OTU264 | k__Bacteria;  p__Bacteroidetes;  c__Flavobacteriia;  o__Flavobacteriales; f__Cryomorphaceae;  g__;  s__ |
| Microeukaryotes | ACF | Connector | OTU1269 | k__Eukaryota;  p__;  c__;  o__;  f__;  g__ |
| Microeukaryotes | ACF | Connector | OTU1541 | k__Eukaryota;  p__;  c__;  o__Choanoflagellida;  f__;  g__ |
| Microeukaryotes | ACF | Connector | OTU32 | k__Eukaryota;  p__;  c__;  o__;  f__ ;  g__Nudifila |
| Microeukaryotes | PVC | Connector | OTU262 | k__Eukaryota  p__Bacillariophyta; c__Bacillariophyceae;  o__Naviculales;  f__Naviculaceae  g__Navicula |
| Microeukaryotes | Pebble | Connector | OTU142 | k__Viridiplantae  p__Streptophyta;  c__rosids;  o__Rosales;  f__Rosaceae  g__Prunus |
| Microeukaryotes | Pebble | Connector | OTU18 | k__Viridiplantae;  p__Chlorophyta;  c__Chlorophyceae;  o__Sphaeropleales;  f__Sphaeropleaceae;  g__Tetraedron |
| Microeukaryotes | Pebble | Connector | OTU19 | k__Viridiplantae;  p__Chlorophyta;  c__Trebouxiophyceae;  o__Chlorellales;  f__;  g__Parachlorella |
| Microeukaryotes | Pebble | Connector | OTU191 | k__Viridiplantae;  p__Streptophyta;  c__Liliopsida;  o__Alismatales;  f__Araceae;  g__Zantedeschia |
| Microeukaryotes | Pebble | Connector | OTU46 | k__Eukaryota;  p__Bacillariophyta; c__Bacillariophyceae;  o__Naviculales;  f__Pinnulariaceae；  g__Pinnularia |
| Microeukaryotes | Wood | Connector | OTU119 | k__Eukaryota；  p__；  c__；  o__Rigifilida；  f__；  g__Micronuclearia |
| Microeukaryotes | Wood | Connector | OTU12 | k__Viridiplantae；  p__Chlorophyta；  c__Chlorophyceae；  o__；  f__；  g__ |
| Microeukaryotes | Wood | Connector | OTU1819 | k__Eukaryota;  p__;  c__;  o__;  f__;  g__ |

**Supplementary Table S13.** The relative contribution of the top 10 metabolic function pathways of bacteria and fungi to the biofilm communities from the four substrates: ACF, PVC, pebble and wood. Significant differences were conducted between artificial (ACF and PVC) and natural (pebble and wood) substrates by performing a one-way ANOVA followed by Tukey’s posthoc tests.

| Metabolic function pathways (%) | | Artificial | | Natural | | 1. values   (Artificial vs Natural) |
| --- | --- | --- | --- | --- | --- | --- |
|  |  | ACF | PVC | Pebble | Wood |  |
| Bacteria | Phototrophy | 14.40 | 19.21 | 19.68 | 13.40 | 0.86236 |
|  | Chemoheterotrophy | 14.37 | 9.78 | 8.83 | 21.99 | 0.17194 |
|  | Aerobic_chemoheterotrophy | 13.52 | 9.14 | 8.37 | 20.97 | 0.15501 |
|  | Photoautotrophy | 9.76 | 14.33 | 15.77 | 7.58 | 0.83634 |
|  | Cyanobacteria | 9.68 | 14.26 | 15.73 | 7.43 | 0.83139 |
|  | Oxygenic_photoautotrophy | 9.68 | 14.26 | 15.73 | 7.43 | 0.83139 |
|  | Photoheterotrophy | 4.71 | 4.95 | 3.95 | 5.97 | 0.8577 |
|  | Intracellular_parasites | 4.20 | 2.27 | 0.97 | 0.83 | 1.35874E-4 |
|  | Fermentation | 1.12 | 0.74 | 1.31 | 2.08 | 0.00234 |
|  | Nitrate_reduction | 1.69 | 0.99 | 0.59 | 0.71 | 0.00169 |
| Fungi | Bryophyte Parasite-Dung Saprotroph -Ectomycorrhizal-Fungal Parasite -Leaf Saprotroph-Plant Parasite -Undefined Saprotroph-Wood Saprotroph | 62.08 | 20.23 | 17.75 | 42.16 | 0.19049 |
|  | Animal Endosymbiont-Animal Pathogen-Plant Pathogen-Undefined Saprotroph | 4.99 | 36.54 | 37.94 | 1.41 | 0.8967 |
|  | Animal Pathogen | 13.91 | 24.56 | 22.40 | 0.99 | 0.13193 |
|  | Plant Pathogen | 4.73 | 5.35 | 11.53 | 39.88 | 1.53558E-4 |
|  | Undefined Saprotroph | 6.60 | 3.96 | 2.35 | 10.50 | 0.52916 |
|  | Lichenized | 2.65 | 3.67 | 4.37 | 1.03 | 0.56325 |
|  | Animal Endosymbiont | 1.61 | 3.64 | 0.53 | 0.77 | 0.0039 |
|  | Plant Saprotroph | 1.47 | 1.19 | 1.89 | 1.34 | 0.49178 |
|  | Epiphyte | 0.34 | 0.35 | 0.22 | 0.30 | 0.64137 |
|  | Plant Pathogen-Undefined Parasite -Undefined Saprotroph | 0.27 | 0.07 | 0.65 | 0.14 | 0.22256 |

**Supplementary Table S14.** The effects of substrate on the function variances assessed using permutational multivariate analyses of variance (PERMANOVA, ‘adonis and anosim’ in vegan R package) with 999 random permutations.

| Paired samples | | Adonis | | | Anosim | |
| --- | --- | --- | --- | --- | --- | --- |
|  |  | F | R^2^ | P | R | P |
| Bacteria | ACF-Pebble | 5.8 | 0.37 | 0.018 | 0.43 | 0.016 |
|  | ACF-PVC | 3.2 | 0.24 | 0.071 | 0.15 | 0.126 |
|  | ACF-Wood | 7.4 | 0.42 | 0.007 | 0.60 | 0.004 |
|  | Pebble-PVC | 0.9 | 0.09 | 0.378 | 0.01 | 0.370 |
|  | Pebble-Wood | 10.4 | 0.51 | 0.004 | 0.77 | 0.002 |
|  | PVC-Wood | 8.8 | 0.47 | 0.001 | 0.73 | 0.002 |
|  | ACF, PVC-Pebble,Wood | 3.8 | 0.15 | 0.010 | 0.19 | 0.016 |
| Fungi | ACF-Pebble | 18.8 | 0.65 | 0.003 | 0.82 | 0.001 |
|  | ACF-PVC | 18.4 | 0.65 | 0.001 | 0.79 | 0.003 |
|  | ACF-Wood | 17.5 | 0.64 | 0.005 | 0.87 | 0.003 |
|  | Pebble-PVC | 1.2 | 0.11 | 0.274 | 0.07 | 0.191 |
|  | Pebble-Wood | 54.1 | 0.84 | 0.004 | 1.00 | 0.002 |
|  | PVC-Wood | 74.3 | 0.88 | 0.003 | 1.00 | 0.002 |
|  | ACF, PVC-Pebble,Wood | 3.1 | 0.12 | 0.069 | 0.17 | 0.031 |

**Supplementary Table S15.** Comparison of the bacterial and fungal metabolic function pathways between the artificial (ACF and PVC) and natural (pebble and wood) substrates by STAMP. Significant differences were determined by Welch’s unequal variances t-test and then corrected for multiple tests according to the Benjamini–Hochberg false discovery rate (FDR) procedure. The q-values of bacteria were lower than 0.01, and those of fungi were lower than 0.05.

| Category | Metabolic function pathways | Artificial: mean rel. freq. (%) | Artificial: std. dev. (%) | Natural: mean rel. freq. (%) | Natural: std. dev. (%) | p-values | p-values (corrected) | Difference between means | 95.0% lower CI | 95.0% upper CI |
| --- | --- | --- | --- | --- | --- | --- | --- | --- | --- | --- |
| Bacteria | Aromatic_compound_degradation | 0.36809375 | 0.119948005 | 0.993963243 | 0.453559356 | 0.000748053 | 0.004692332 | -0.625869493 | -0.932630016 | -0.319108971 |
|  | Arsenate_respiration | 0.936003408 | 0.455781528 | 0.197682805 | 0.075273364 | 0.000210866 | 0.003637446 | 0.738320603 | 0.433679163 | 1.042962043 |
|  | Dissimilatory_arsenate_reduction | 0.936003408 | 0.455781528 | 0.197682805 | 0.075273364 | 0.000210866 | 0.004849928 | 0.738320603 | 0.433679163 | 1.042962043 |
|  | Human_gut | 0.492637022 | 0.14140811 | 1.069012984 | 0.452703623 | 0.001401151 | 0.007436878 | -0.576375962 | -0.885005248 | -0.267746675 |
|  | Intracellular_parasites | 3.236450026 | 1.665712306 | 0.900597524 | 0.220723782 | 0.000687217 | 0.00592725 | 2.335852503 | 1.22538339 | 3.446321615 |
|  | Mammal_gut | 0.525472994 | 0.157271752 | 1.085784865 | 0.448769173 | 0.00164646 | 0.008114698 | -0.560311871 | -0.868541727 | -0.252082014 |
|  | Methanol_oxidation | 0.190359961 | 0.086229561 | 0.077989757 | 0.02618136 | 0.00117025 | 0.006728937 | 0.112370204 | 0.053675508 | 0.171064901 |
|  | Methanotrophy | 0.067857909 | 0.025283632 | 0.029448186 | 0.012580501 | 0.000348624 | 0.004009175 | 0.038409724 | 0.020371043 | 0.056448404 |
|  | Methylotrophy | 0.258217871 | 0.080941063 | 0.107437943 | 0.024368885 | 5.14E-05 | 0.003543775 | 0.150779928 | 0.095709695 | 0.205850161 |
|  | Nitrate_ammonification | 0.936003408 | 0.455781528 | 0.197682805 | 0.075273364 | 0.000210866 | 0.002909957 | 0.738320603 | 0.433679163 | 1.042962043 |
|  | Nitrate_respiration | 1.179714703 | 0.575142226 | 0.381888116 | 0.084554589 | 0.000743449 | 0.005129797 | 0.797826587 | 0.413987405 | 1.181665768 |
|  | Nitrite_ammonification | 0.949568295 | 0.459683762 | 0.204261922 | 0.07514556 | 0.000209546 | 0.007229324 | 0.745306373 | 0.438102297 | 1.05251045 |
|  | Nitrite_respiration | 1.031941152 | 0.477550419 | 0.307358594 | 0.064743261 | 0.000368712 | 0.003634451 | 0.724582558 | 0.406148019 | 1.043017097 |
|  | Nitrogen_respiration | 1.190918097 | 0.578679106 | 0.388292361 | 0.087019294 | 0.000743405 | 0.00569944 | 0.802625735 | 0.416323166 | 1.188928304 |
|  | Reductive_acetogenesis | 0.033394801 | 0.016576775 | 0.013194532 | 0.006605837 | 0.00203889 | 0.009378893 | 0.020200269 | 0.008691185 | 0.031709352 |
| Fungi | Plant Pathogen | 5.041632642 | 1.50842184 | 25.70570916 | 14.95215138 | 0.000775172 | 0.014728266 | -20.66407652 | -30.61280156 | -10.71535147 |

**

**

**Supplementary Figure S1.** The growth curve of biofilms on artificial (ACF and PVC) and natural (pebble and wood) substrates, and the detailed values are shown in Supplementary Table S4.


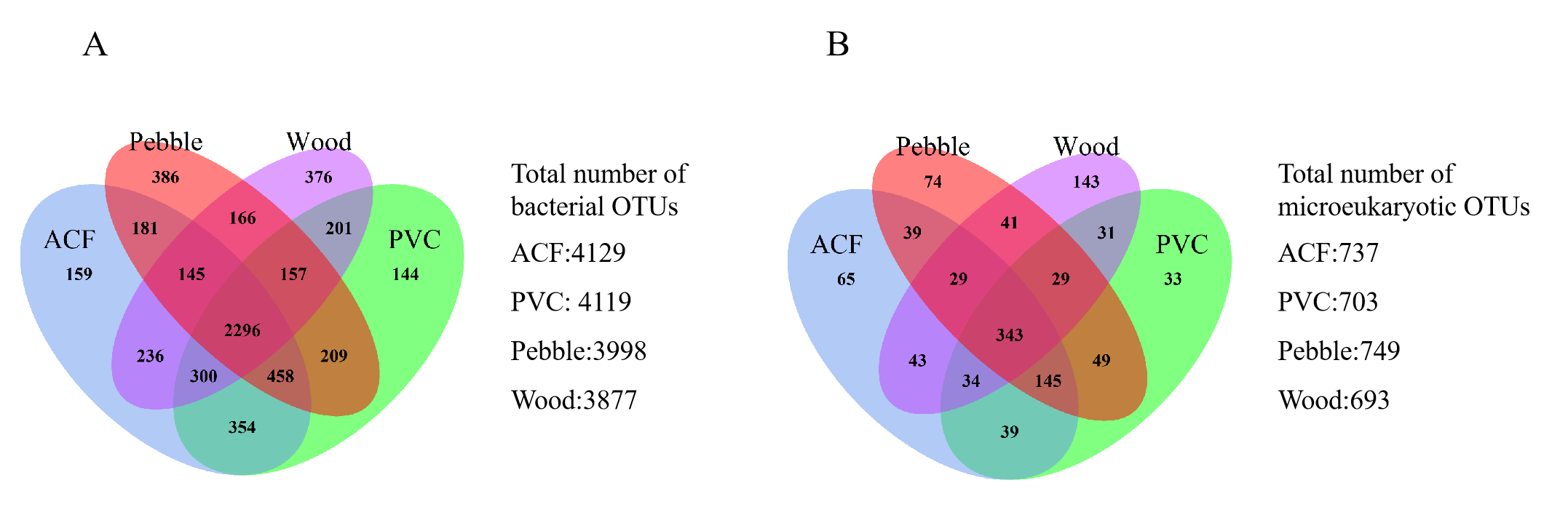
**Supplementary Figure S2.** Venn diagrams display the number of shared and unique OTUs of biofilms of bacteria (A) and microeukaryotes (B) from artificial (ACF and PVC) and natural (pebble and wood) substrates.

**Supplementary Figure S3.** Alpha diversity of bacteria including observed species (A) and Shannon (B) indices and microeukaryotes composed of observed species (C) and Shannon (D) indices, for artificial (ACF and PVC) and natural (pebble and wood) substrates. And the letters represent a significant difference at p-value < 0.05 by using one-way ANOVA followed by Tukey's posthoc tests.

**Supplementary Figure S4.** PCoA plots depict bacterial (A) and microeukaryotic (B) biofilms on artificial (ACF and PVC) and natural (pebble and wood) substrates using the phylogenetically weighted UniFrac distance matrix. Statistical comparison between different samples was deduced by PERMANOVA, and results are provided in Supplementary Table S6.


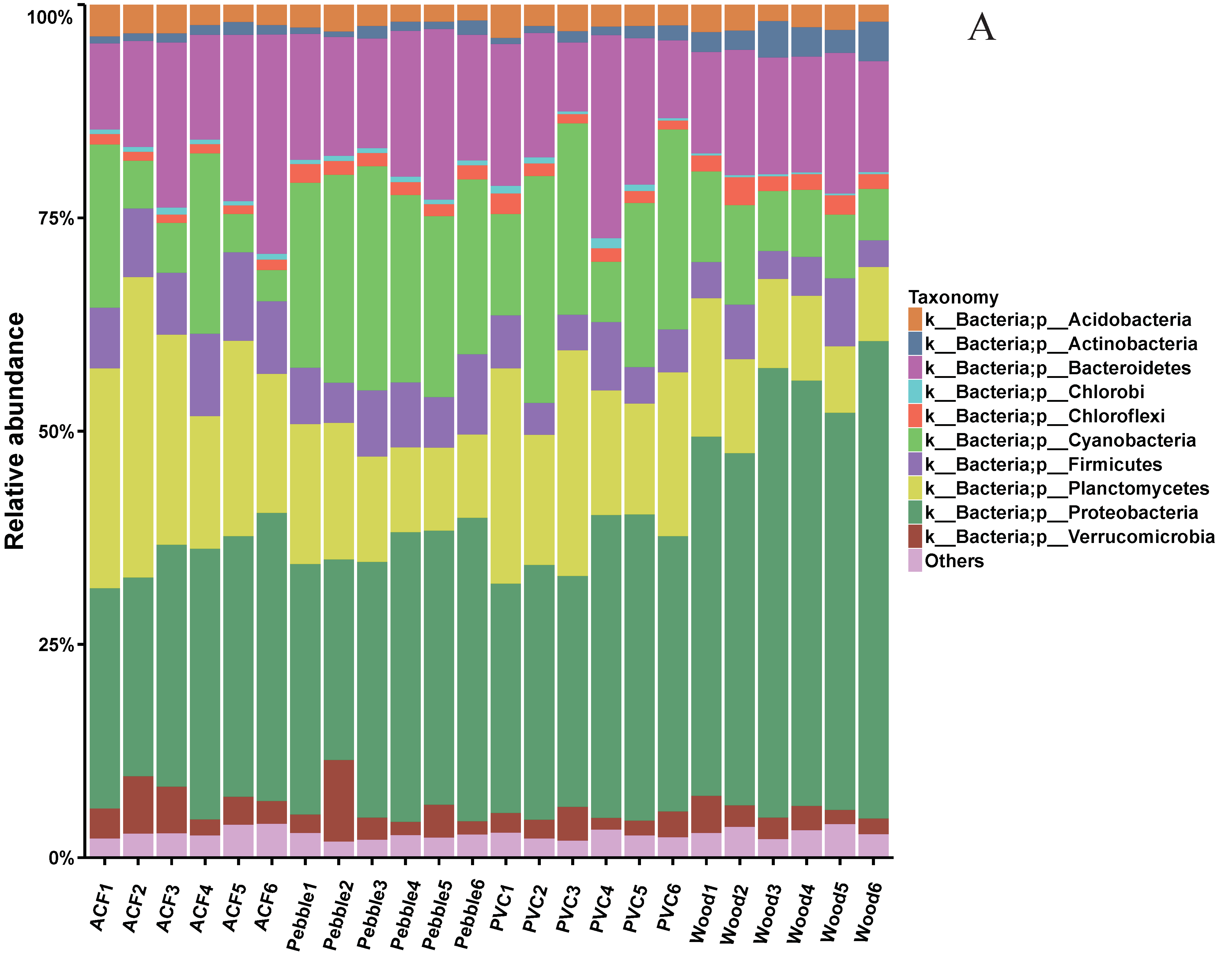

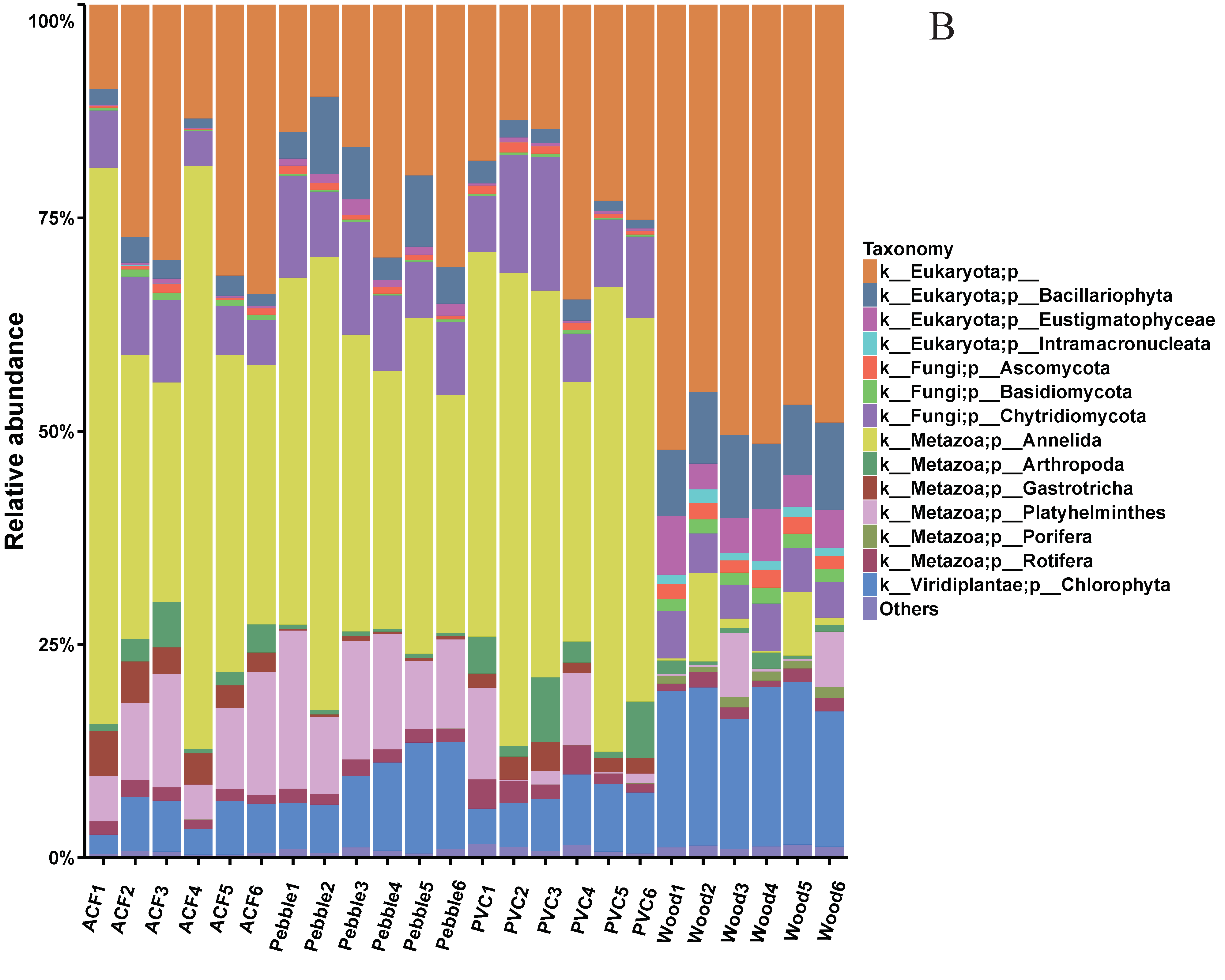


**Supplementary Figure S5.** The distribution of relative abundance at phylum level of bacteria (A) and microeukaryotes (B) from artificial (ACF and PVC) and natural (pebble and wood) substrates (n=6). Statistical analysis between the substrate types was performed using one-way analysis of variance followed by Tukey's posthoc tests, and results are provided in Supplementary Table S7.


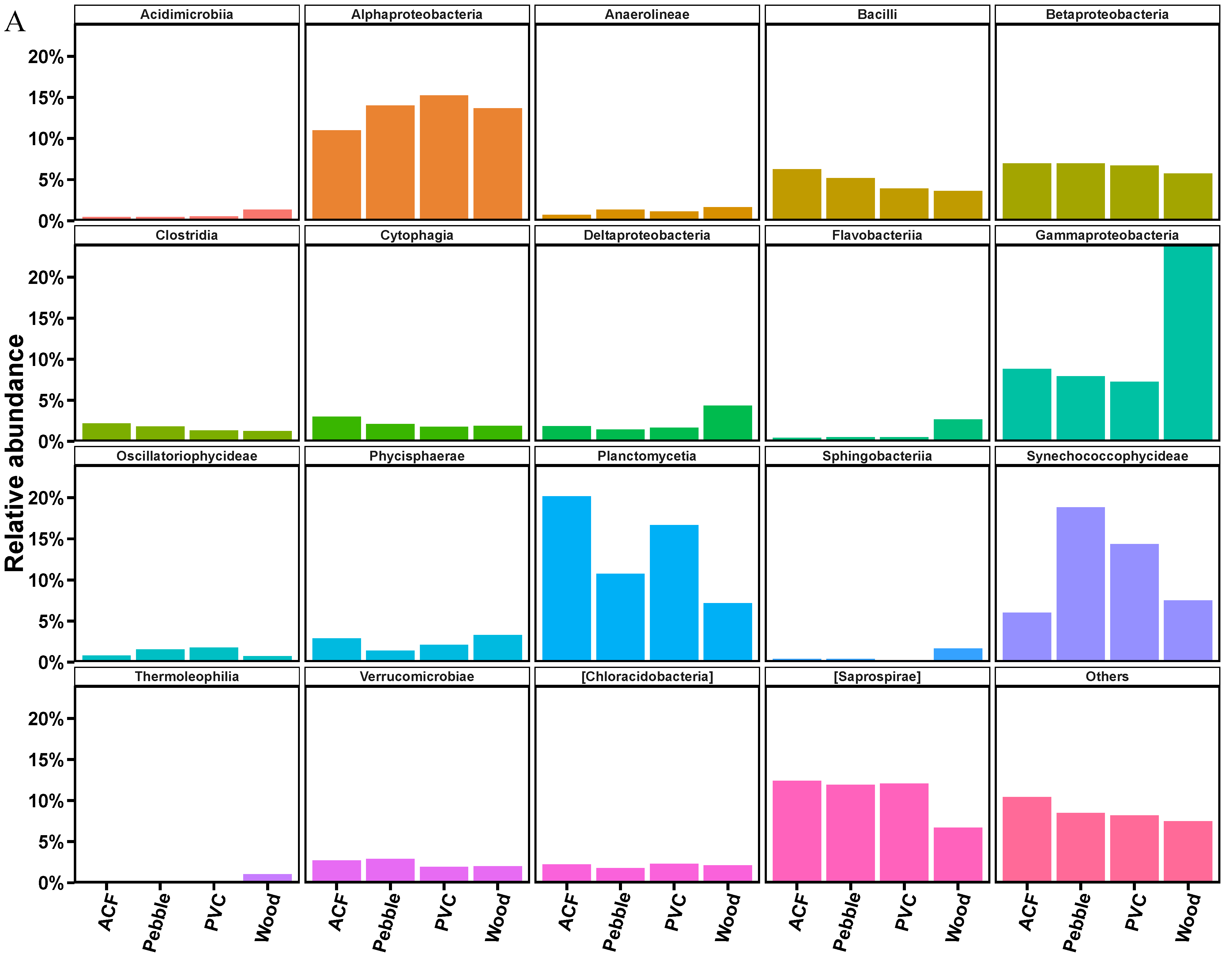


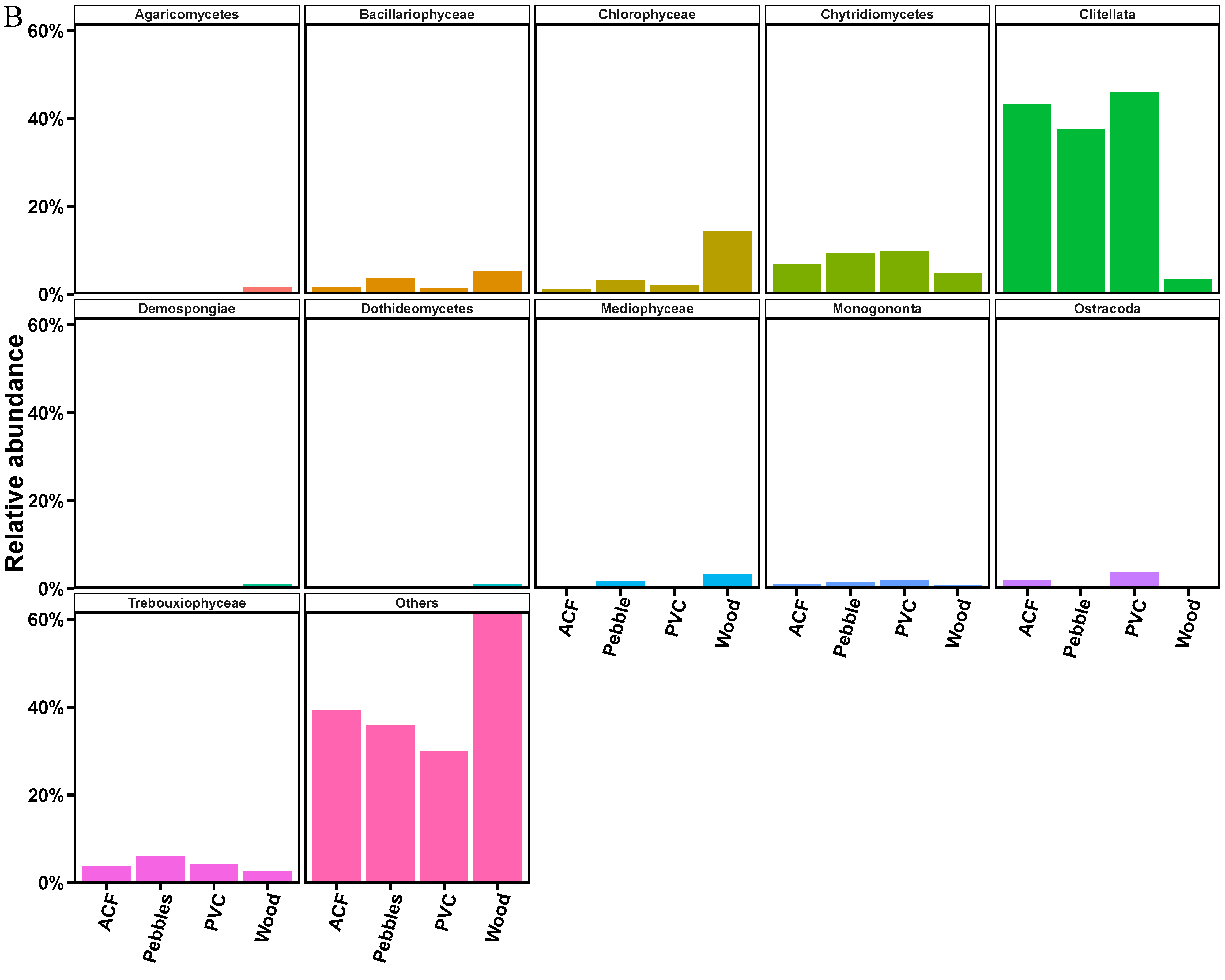


**Supplementary Figure S6.** (A)Relative abundance of the top 20 most abundant classes in bacterial biofilms from artificial (ACF and PVC) and natural (pebble and wood) substrates.(B) Relative abundance of the top 12 most abundant classes in microeukaryotic biofilms from artificial (ACF and PVC) and natural (pebble and wood) substrates. Statistical analysis between the substrate types was performed using one-way analysis of variance followed by Tukey's posthoc tests, and results are provided in Supplementary Table S8.


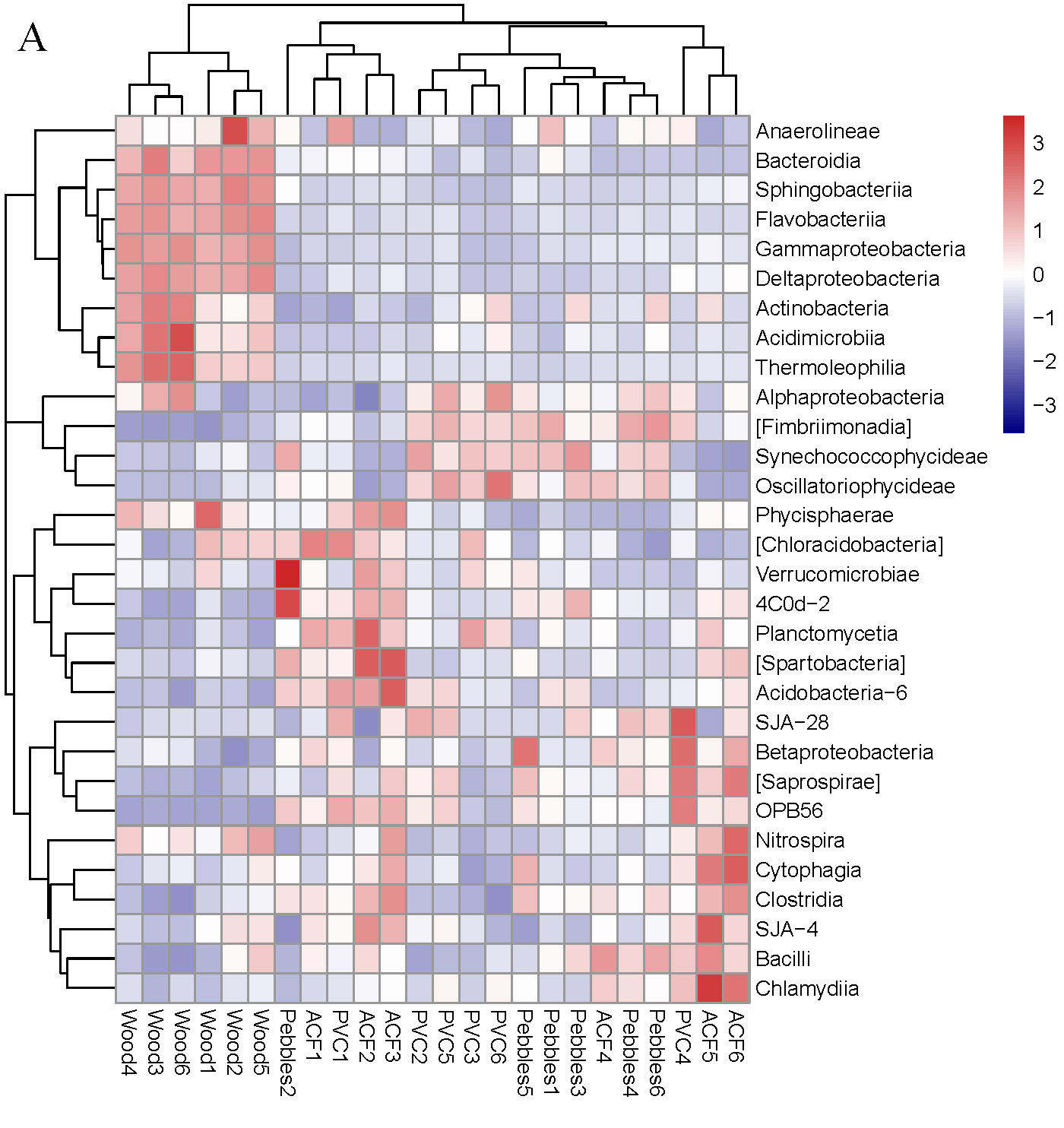


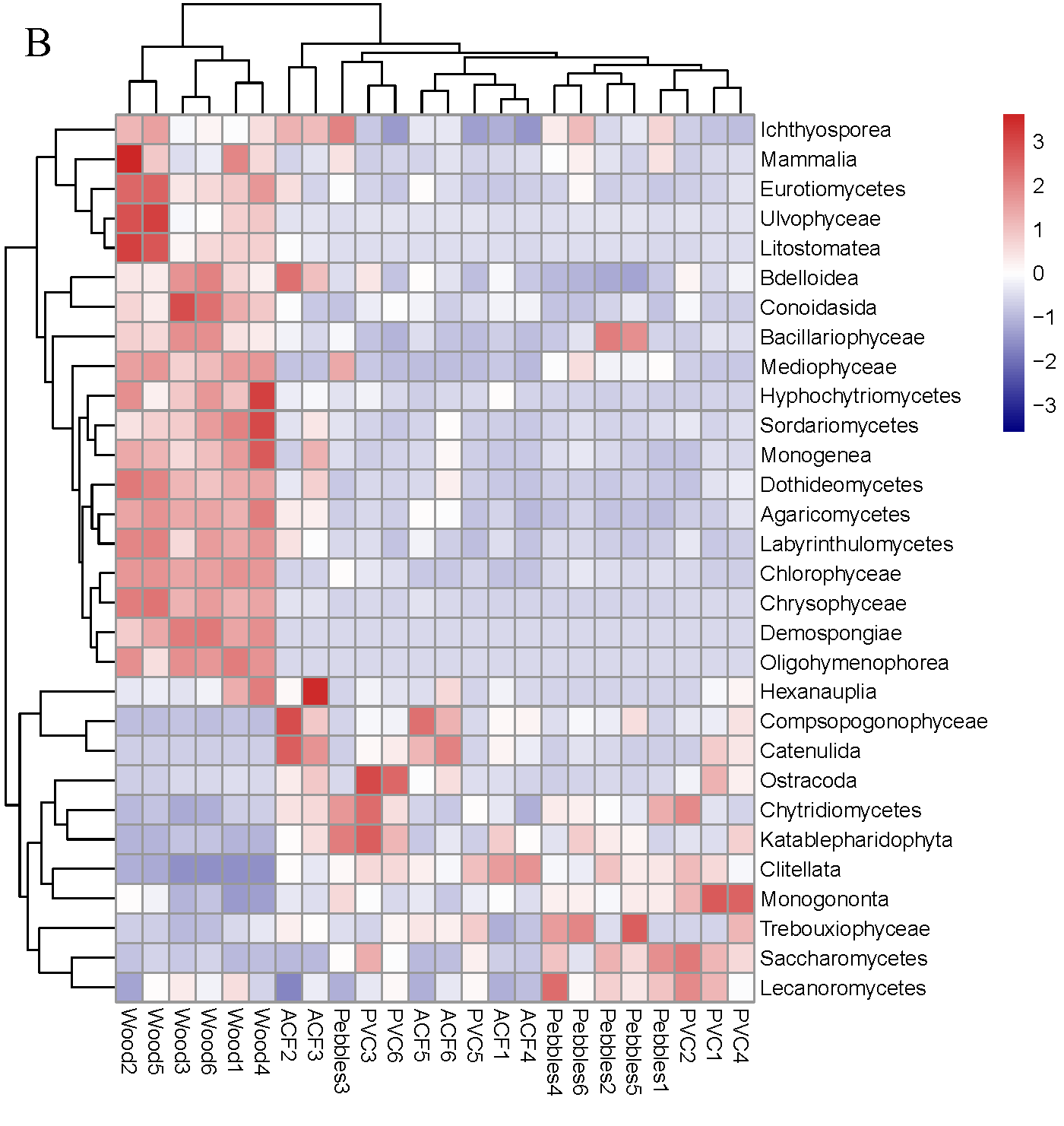


**Supplementary Figure S7.** (A)Heatmap analysis of bacterial biofilms at the class level from artificial (ACF and PVC) and natural (pebble and wood) substrates (n=6).(B)Heatmap analysis of microeukaryotic biofilms at the class level from artificial (ACF and PVC) and natural (pebble and wood) substrates (n=6).

**
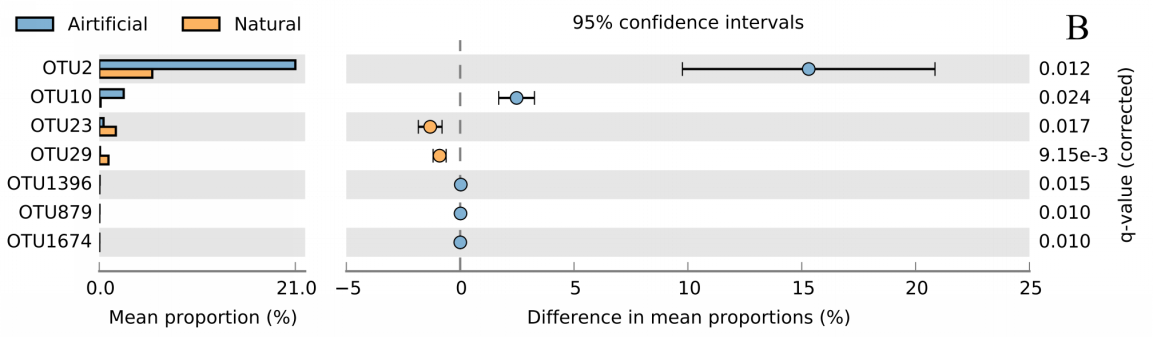

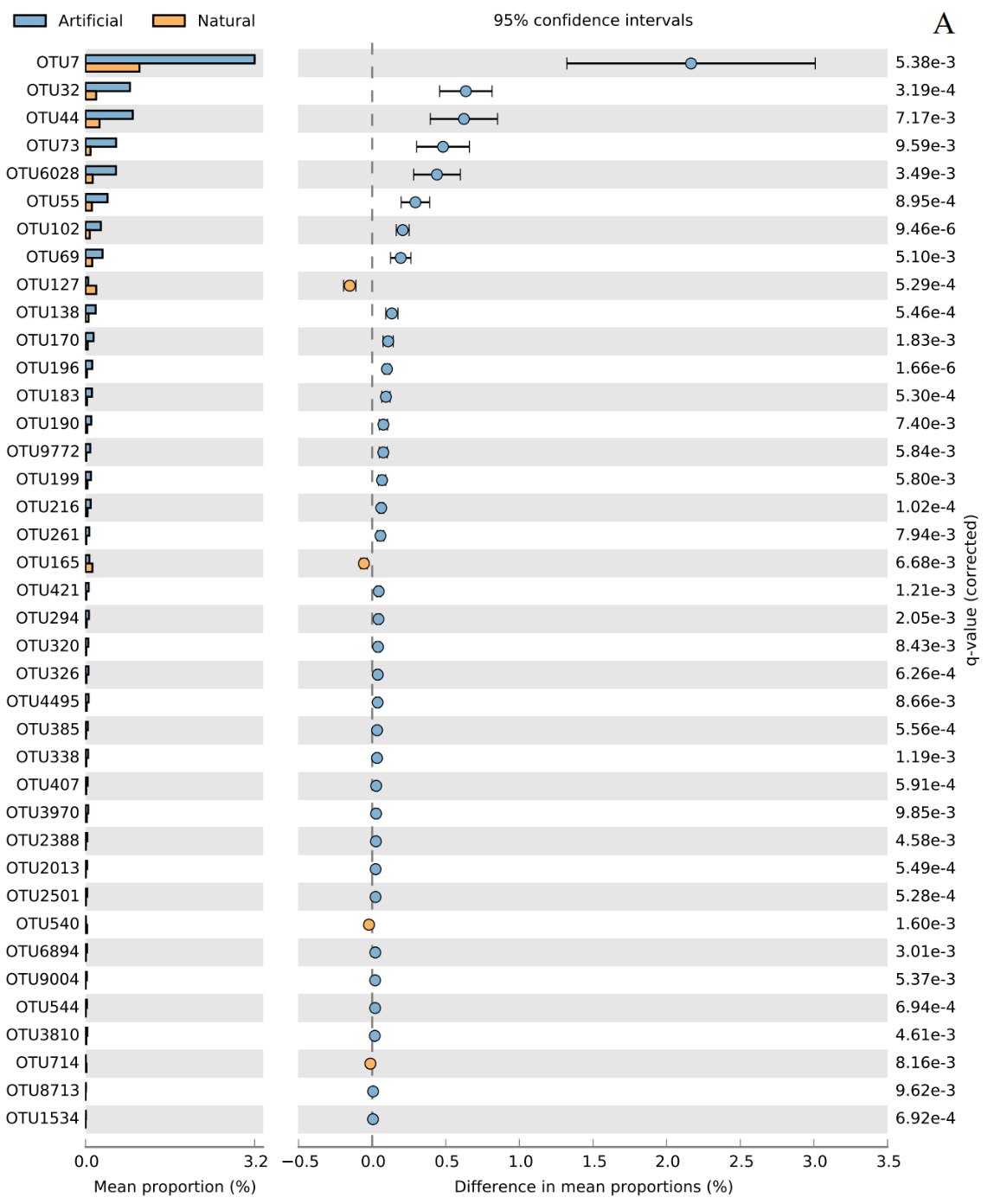
Supplementary Figure S8.** Comparison of the bacterial (A) and microeukaryotic (B) OTU abundance between artificial (ACF and PVC) and natural (pebble and wood) substrates. Significant differences were determined by Welch’s unequal variances t-test and then corrected for multiple tests according to the Benjamini–Hochberg false discovery rate (FDR) procedure. The q-values of bacteria were lower than 0.01, and those of microeukaryotes were lower than 0.05.See also Supplementary Table S9.

**Supplementary Figure S9.** (A)Relative abundance of the top 10 most abundant metabolic function pathways in fungal biofilms from artificial (ACF and PVC) and natural (pebble and wood) substrates. Statistical analysis between the substrate types was performed using one-way analysis of variance followed by Tukey's posthoc tests, and results are provided in Supplementary Table S13. (B) Heatmap-mean analysis of fungal biofilms at the function level from artificial (ACF and PVC) and natural (pebble and wood) substrates (n=6). (C)PCA plot depicts fungal biofilms on artificial (ACF and PVC) and natural (pebble and wood) substrates based on the Bray-Curtis distance. Statistical comparison between different samples was deduced by PERMANOVA, and results are provided in Supplementary Table S14. (D) Comparison of the fungal metabolic function pathways’ abundance between artificial (ACF and PVC) and natural (pebble and wood) substrates. Significant differences were determined by Welch’s unequal variances t-test and then corrected for multiple tests according to the Benjamini–Hochberg false discovery rate procedure. The q-values of fungi were lower than 0.05. See also Supplementary Table S15.
